# Beyond carbon dioxide: decoupling headspace and buffering reveals a bicarbonate requirement for anaerobic gut fungi growth

**DOI:** 10.1186/s40694-026-00224-0

**Published:** 2026-10-07

**Authors:** Kevin Edward Schulz, Alisa Pfau, Tuba Tarhan, Charlotte van Almsick, Dominik Scholz, Anto Rafael Sikirić, Anke Neumann, Katrin Ochsenreither

**Affiliations:** 1 Karlsruhe Institute of Technology, Fritz-Haber-Weg 4, 76131 Karlsruhe, Germany; 2 Kaiserslautern University of Applied Sciences, Carl-Schurz-Str. 10 – 16, 66953 Pirmasens, Germany

**Keywords:** *Neocallimastigomycota*, Hydrogenosomal metabolism, Bicarbonate requirement, Inorganic carbon availability, Anaplerotic metabolism, Gas-phase effects, Biomass quantification

## Abstract

**Background:**

Anaerobic gut fungi (*Neocallimastigomycota*) are major lignocellulose degraders in herbivore gastrointestinal tracts, but reproducible axenic cultivation remains difficult because conventional serum-bottle systems intrinsically couple CO_2_ headspace, bicarbonate buffering, dissolved inorganic carbon availability, and pH control. This study aimed to decouple these variables and determine whether bicarbonate is required beyond its buffering function.

**Results:**

Comparison of CO_2_- and N_2_-based headspaces in otherwise identical bicarbonate-buffered cultures showed consistently higher hydrogen production under N_2_. Screening of chemically defined non-carbonate buffers demonstrated that stable external pH alone was insufficient to sustain activity, as bicarbonate-free cultures lost metabolic performance after serial transfer. Reintroduction of sodium hydrogen carbonate under N_2_ restored activity in a strain-dependent manner, revealing low bicarbonate thresholds that supported reproducible growth-associated metabolism in filamentous taxa. To test whether this effect reflected a general carboxylate requirement, twenty structurally diverse carboxylic acids were screened using *Aestipascuomyces dupliciliberans*; none restored the bicarbonate-supported hydrogen production phenotype, although maleic acid increased soluble metabolite accumulation. A dual-buffer strategy combining Bis-Tris with minimal bicarbonate enabled independent pH control and identified maximal metabolic activity at pH 6.4. In addition, an OD_750_-based biomass assay calibrated with activated carbon was established and validated in an ammonium sulfate perturbation experiment, providing a practical framework for biomass estimation and future analyses of nitrogen effects.

**Conclusions:**

Headspace composition and inorganic carbon availability are key determinants of anaerobic gut fungal cultivation. The results indicate that bicarbonate supports sustained anaerobic gut fungal activity beyond extracellular pH stabilization and cannot be functionally replaced by the tested organic carboxylates. Decoupling gas phase, buffering chemistry, and pH control provides a more interpretable and reproducible cultivation framework for physiological studies and biotechnological application of anaerobic gut fungi.

**Supplementary Information:**

The online version contains supplementary material available at https://doi.org/10.1186/s40694-026-00224-0.

## Background

Anaerobic gut fungi (AGF), classified within the phylum *Neocallimastigomycota*, are obligate anaerobic eukaryotes inhabiting the gastrointestinal tracts of herbivorous vertebrates, including mammals and reptiles [1, 2]. Within the rumen ecosystem, AGF function as primary colonizers of lignocellulosic plant material. Through invasive rhizoidal growth and the formation of appressorium-like structures, AGF penetrate intact plant tissues, physically disrupt recalcitrant cell wall architecture, and thereby enhance enzymatic accessibility [1, 3]. In parallel, AGF secrete a broad spectrum of carbohydrate-active enzymes (CAZymes), frequently organized into fungal cellulosome-like multi-enzyme complexes that mediate efficient polysaccharide depolymerization [4, 5]. Through combined mechanical and enzymatic strategies, AGF substantially contribute to fiber degradation and carbon turnover in anaerobic digestive systems.

Beyond their ecological relevance, AGF have attracted increasing attention for biotechnological exploitation, particularly in the context of lignocellulose valorization and biological hydrogen production [6, 7]. Hydrogen formation in AGF occurs within hydrogenosomes, mitochondria-derived organelles that couple pyruvate oxidation to molecular hydrogen production via reduced ferredoxin-dependent hydrogenases [8]. Because hydrogen represents a direct fermentation end product and a key determinant of redox balance, cultivation conditions that influence hydrogen partial pressure and intracellular carbon flux are expected to strongly affect metabolic performance.

Despite this potential, reproducible *axenic* cultivation of AGF remains technically challenging. Current cultivation strategies are largely derived from the classical Hungate serum bottle technique [9] and typically rely on CO_2_-containing headspaces combined with bicarbonate-based buffering systems [7]. While this setup approximates ruminal conditions, it introduces an intrinsic physicochemical coupling between atmospheric composition, carbonate equilibria, dissolved gas concentrations, and medium pH. During batch cultivation in closed systems, metabolic production of H_2_ and CO_2_ increases headspace pressure, thereby altering gas solubility and shifting fermentation equilibria. In anaerobic fermentative systems, hydrogen partial pressure directly influences metabolic flux partitioning and end-product distribution [10]. Simultaneously, the CO_2_/HCO_3_⁻ equilibrium determines extracellular buffering capacity and pH stability. Consequently, atmospheric composition, the buffer system, and metabolic activity become interdependent variables, complicating mechanistic interpretation and reducing reproducibility.

Although earlier studies demonstrated that modified gas compositions can alter fermentation patterns in rumen fungi [10], these investigations did not systematically decouple headspace composition from carbonate-based buffers. Notably, even studies that include N_2_ in the headspace often retain substantial CO_2_ fractions (typically 30–35%) and/or maintain bicarbonate in the medium, such that carbonate equilibria remain functionally coupled to gas-phase composition [10–13]. Thus, the independent contribution of atmospheric composition versus soluble carbon buffers has not been resolved experimentally. Because bicarbonate fulfills a dual role, acting both as a pH buffer and as a participant in inorganic carbon equilibria, alterations in gas phase composition inherently modify medium chemistry. Nitrogen, in contrast, does not participate in carbonate equilibria under cultivation conditions and therefore enables experimental isolation of headspace-dependent effects. From a practical perspective, N_2_-based cultivation may also simplify gas handling, because nitrogen can be generated on site using commercially established pressure-swing adsorption or membrane systems, thereby reducing dependence on premixed compressed-gas cylinders and potentially lowering gas-supply costs [14–16]. Establishing N_2_-based headspace conditions therefore provides a defined experimental framework to isolate atmospheric redox effects independently of CO_2_-driven buffering interactions.

However, atmospheric decoupling alone is insufficient if buffering chemistry remains intrinsically linked to carbonate equilibria. To isolate physicochemical from physiological effects, bicarbonate must be replaced by chemically defined, CO_2_-independent buffer systems. Zwitterionic Good’s buffers, designed to minimize biological reactivity and metal interactions [17], offer a suitable alternative for stable pH control independent of carbonate dynamics. This strategy enables independent manipulation of headspace composition and medium pH.

Yet, substitution of bicarbonate raises a fundamental physiological question. In many anaerobic systems, dissolved inorganic carbon is not merely a buffering component but functions as a substrate for anaplerotic carboxylation reactions, replenishing oxaloacetate and maintaining carbon flux through central metabolism [18, 19]. Such CO_2_/HCO_3_⁻ dependencies are not restricted to strictly anaerobic microorganisms. Carbon dioxide availability can also affect the growth of facultative and aerobic heterotrophic bacteria at low CO_2_ concentrations, as shown for *Escherichia coli* under CO_2_-limited conditions [20]. In anaerobic gut fungi, cultivation has historically required CO_2_-containing atmospheres and bicarbonate supplementation [7, 21], suggesting that inorganic carbon availability may support metabolic functions beyond extracellular pH stabilization. Whether this reflects a direct metabolic requirement of hydrogenosomal or central carbon metabolism, or merely a physicochemical dependency of conventional cultivation systems, remains unresolved. Consequently, it is unclear whether bicarbonate acts solely as a buffering agent or as a physiologically enabling factor for sustained fungal growth. Whether alternative organic carboxylates can functionally replace this role remains unresolved.

Resolving these questions requires cultivation conditions in which atmospheric composition, buffering chemistry, and inorganic carbon availability can be manipulated independently. At the same time, interpretation of metabolic responses requires an approximate distinction between changes in metabolic flux and changes in biomass formation. This remains challenging because anaerobic gut fungi form complex rhizomycelial networks and strongly associate with particulate substrates, limiting the applicability of conventional biomass quantification methods [7, 11]. Consequently, changes in fermentation products cannot readily be attributed to altered metabolic activity per unit biomass or to differences in fungal growth. Furthermore, although ammonium sulfate is routinely supplied as the principal nitrogen source in defined cultivation media [7], comparatively little is known about whether moderate variation in nitrogen availability measurably affects growth and metabolism relative to inorganic carbon availability. Addressing these methodological and physiological knowledge gaps is essential for establishing reproducible cultivation strategies and for enabling mechanistic investigations of anaerobic gut fungal physiology.

To address these questions, this study establishes an experimental framework that progressively decouples atmospheric composition, buffering chemistry, inorganic carbon availability, and nutrient supply during anaerobic gut fungal cultivation. This framework was used to determine whether bicarbonate is required beyond its buffering function and to evaluate the independent contributions of headspace composition, pH, and inorganic carbon availability to fungal metabolism. Because biomass determination remains a major limitation in physiological studies of anaerobic gut fungi, an OD_750_-based biomass proxy was additionally developed to facilitate interpretation of metabolic responses. Finally, an initial nitrogen-variation experiment was included to examine whether moderate changes in ammonium availability measurably influence growth and metabolism under defined cultivation conditions. Based on the long-standing requirement for CO_2_-containing atmospheres and bicarbonate supplementation in anaerobic gut fungal cultivation, we hypothesized that inorganic carbon availability would exert a stronger influence on metabolic performance than moderate variation in nitrogen availability within the tested concentration range. By experimentally separating these interacting variables, this study aims to establish a more mechanistically interpretable and reproducible cultivation framework for physiological and biotechnological investigations of anaerobic gut fungi.

## Methods

The experimental procedures were adapted from previously established anaerobic gut fungi cultivation and analytical workflows [22]. All methods are described in full below to ensure independent reproducibility. Any deviations from the referenced protocol are explicitly indicated in the corresponding sections.

### Fungal strains

The anaerobic gut fungal strains used in this study were *Aestipascuomyces dupliciliberans* A252, *Caecomyces churrovis* PP313, *Khoyollomyces ramosus* X2152, *Orpinomyces joyonii* W212, *Pecoramyces ruminantium* SA222, and *Neocallimastix cameroonii* G341. All strains belong to the phylum *Neocallimastigomycota* and were originally isolated from herbivore dung samples and taxonomically characterized as described by Stabel et al. [23]. Strains were maintained under standardized batch cultivation conditions with a seven-day transfer cycle at 39 °C. Unless otherwise stated, inoculation was performed by transferring 5 mL of a 7-day preculture into 50 mL of fresh medium. Prior to transfer, cultures were gently homogenized by manual agitation to disrupt rhizoidal aggregates and ensure comparable biomass distribution within the inoculum. For mechanistic analyses focusing on atmospheric effects, bicarbonate dependency, and metabolic flux distribution, experiments were primarily conducted using filamentous anaerobic gut fungi (*A. dupliciliberans*,* O. joyonii*,* P. ruminantium*,* N. cameroonii).* Filamentous strains form extensive rhizoidal networks and exhibit robust hydrogenosomal activity, providing greater experimental stability under perturbation conditions. Bulbous taxa such as *C. churrovis* and bulbous-like filamentous taxa like *K. ramosus* were included in the initial atmospheric screening and in biomass quantification experiments to account for strain variability. Preliminary experiments indicated reduced growth stability of bulbous strains under strictly carbonate-independent conditions; therefore, subsequent mechanistic experiments were restricted to filamentous taxa.

### Cultivation medium

The minimal cultivation medium used in this study was based on the formulation established by Schulz et al. [22], with modifications as indicated in the respective experimental sections. The complete composition is provided here to ensure reproducibility.

Per liter of medium, 150 mL of salt solution I (3 g L^− 1^ dipotassium hydrogen phosphate in demineralized water) and 150 mL of salt solution II (3 g L^− 1^ potassium dihydrogen phosphate, 6 g L^− 1^ ammonium sulfate, 6 g L^− 1^ sodium chloride, 0.6 g L^− 1^ magnesium sulfate heptahydrate, and 0.6 g L^− 1^ calcium chloride dihydrate in demineralized water) were combined. To support zoospore formation and anaerobic redox control, 2 mL of a hemin solution (0.5 g L^− 1^ hemin chloride dissolved in a 1:1 mixture of ethanol and 0.05 M NaOH) and 2 mL of a resazurin solution (1 g L^− 1^ resazurin sodium salt in demineralized water) were added. Depending on the experimental setup, demineralized water was added to reach a final mass of 990 g for non-soluble carbon source cultivation or 890 g when soluble carbon sources were used. Unless otherwise specified, sodium hydrogen carbonate (6 g L^− 1^) was included as buffering agent, L-cysteine hydrochloride monohydrate (1 g L^− 1^) was added as reducing agent, and 10 mL L^− 1^ of a trace element solution was supplemented. The trace element solution contained (per liter of 0.2 M HCl): 0.25 g manganese (II) chloride tetrahydrate, 0.25 g nickel(II) chloride hexahydrate, 0.25 g sodium molybdate dihydrate, 0.25 g boric acid, 0.2 g iron(II) sulfate heptahydrate, 0.05 g cobalt(II) chloride hexahydrate, 0.05 g sodium selenite pentahydrate, 0.05 g sodium metavanadate, 0.025 g zinc sulfate, and 0.0319 g copper(II) sulfate pentahydrate. Unless stated otherwise, milled wheat straw (particle size 1 mm; Waldhof Qualitätsstroh, Landwirtschaftlicher Betrieb Uwe Hoferichter, Germany) was used as solid carbon source at a final concentration of 5 g L^− 1^ and was placed directly into serum bottles prior to sterilization. Soluble carbon sources were sterilized separately and added after autoclaving. After adjustment to pH 6.9 with 4 M NaOH or 4 M phosphoric acid, the medium was dispensed as 50 mL aliquots into 100 mL serum bottles. The bottles were sealed with butyl rubber stoppers and aluminum crimp caps and sterilized by autoclaving. Unless otherwise specified, media were prepared in Fernbach flasks and flushed with 100% CO_2_ prior to distribution to ensure anaerobic conditions. When CO_2_ flushing was applied, the medium was sparged until the resazurin indicator turned colorless and was subsequently pH-adjusted under continued CO_2_ flow to stabilize the target pH. When N_2_-based cultivation was performed, anaerobiosis was established by 20 sequential evacuation and nitrogen overpressure cycles using a custom-built gasing station (Figure S1). Each cycle consisted of vacuum application followed by nitrogen pressurization to ensure complete removal of residual oxygen prior to incubation. Immediately before inoculation, each bottle was supplemented with 0.5 mL vitamin solution (not made anaerobic, containing 0.01 mg/L thiamine hydrochloride, 0.652 g/L panthothenic acid hemicalium salt, 0.6 g/L nicotinic acid, 0.02 g/L cyanocobalamin, 0.2 g/L biotin, 0.012 g/L pyridoxine hydrochloride, 0.05 g/L folic acid, and 0.05 g/L P-aminobenzoic acid 0.2 g/L riboflavin) and 0.5 mL antibiotic solution (ampicillin sodium salt, streptomycin sulfate, and penicillin sodium salt at 50 g L^− 1^ each). Vitamin and antibiotic stock solutions were prepared and sterile-filtered according to Schulz et al. (2025); when not stated otherwise, supplementation volumes were kept constant across all experiments to avoid dilution effects. Unless explicitly stated otherwise, no additional reductants (e.g., sulfide) were used beyond L-cysteine hydrochloride monohydrate.

### Experimental setups

#### Gas atmosphere: CO_2_ versus N_2_

To systematically evaluate the influence of atmospheric composition on anaerobic gut fungi cultivation, parallel serum bottle cultures were established under either a CO_2_- or N_2_-based headspace while maintaining identical medium composition and buffering conditions. The bicarbonate buffer system (6 g L^− 1^ NaHCO_3_) was retained in both treatments to isolate headspace effects from buffer substitution. For the N_2_ condition, sealed serum bottles were rendered anaerobic using a custom-built gasing unit applying 20 sequential evacuation and 100% nitrogen overpressure cycles (approximately 2 min per cycle). This procedure resulted in removal of dissolved CO_2_ from the bicarbonate equilibrium system and caused a reproducible alkaline pH shift. Immediately after completion of the N_2_ cycles, pH was determined by aseptically withdrawing a 3 mL sample under minimal atmospheric exposure. The measured pH value (typically 7.5 at room temperature) was used as the reference for both treatments. For the CO_2_ condition, bottles were made anaerobic using 100% CO_2_ and subsequently adjusted with 5 M NaOH to match the pH measured in the N_2_-treated bottles. Thus, both treatments were initiated at identical pH values (pH 7.5), reflecting the equilibrium state of the bicarbonate system after CO_2_ removal in the N_2_ condition. This approach ensured that headspace composition differed exclusively in gas phase chemistry while medium pH was matched between treatments. All conditions were performed in biological triplicates across three independent weekly batches (*n* = 9 per strain and condition). Cultures were incubated at 39 °C for seven days.

#### Buffer system evaluation

Following the isolated assessment of atmospheric effects, a second experimental series investigated the impact of buffer substitution under a 100% N_2_ headspace. To eliminate the intrinsic coupling between CO_2_ and bicarbonate chemistry, all buffer screening experiments were conducted under nitrogen while systematically replacing the bicarbonate buffer system. The minimal medium described in Sect. 2.2 was prepared without sodium hydrogen carbonate for all buffer substitution experiments unless explicitly stated otherwise. Parallel maintenance cultures were continued under standard cultivation conditions containing 6 g L^− 1^ NaHCO_3_ and a CO_2_ headspace to confirm strain viability during the buffer-screening experiment; these cultures were not included in Fig. 3 because they did not represent N_2_-treated bicarbonate-free buffer conditions. Chemically defined non-carbonate buffering systems were evaluated at discrete concentration levels (0.5–10 g L^− 1^) with the specific buffer concentrations listed in Table S1: MOPS, MOPSO, BES, PIPES, ACES, Bis-Tris, Bis-Tris-Propan, phosphate buffer, and citrate–phosphate buffer. After addition of the respective buffering system all bottles were rendered anaerobic using 100% N_2_ via repeated evacuation and overpressure cycles before autoclaving (121 °C, 20 min). Buffering capacity ranges of the tested systems are summarized in Figure S2. For each condition, medium pH was adjusted to 6.9 at room temperature prior to the establishment of anaerobic conditions using 5 M NaOH or 4 M H_3_PO₄. After sterilization and cooling to room temperature, pH was re-measured. Only buffer–concentration combinations maintaining a post-sterilization pH of 6.9 ± 0.1 were considered sufficiently stable and selected for biological evaluation. Because inoculation was performed using precultures maintained under standard NaHCO_3_/CO_2_ conditions, transfer-associated carryover of dissolved inorganic carbon could not be fully excluded during the first cultivation cycle. To minimize potential carryover effects, strains were propagated in a consecutive transfer cycle under the respective N_2_-buffer condition before growth stability was evaluated. Conclusions regarding buffer compatibility were based on sustained hydrogen production and metabolite formation observed after serial transfer rather than on the initial adaptation cycle. Buffer stability screening and initial biological evaluation were performed using the model organism strain *N. cameroonii*, selected due to its robust growth and consistent hydrogen production under standard conditions. All conditions were conducted in biological triplicate. Cultures were incubated at 39 °C for seven days under otherwise identical conditions. Growth performance was assessed by headspace pressure development, hydrogen formation via µGC, liquid metabolite profiling via HPLC, and microscopic verification of rhizomycelial network formation.

#### Determination of minimal bicarbonate levels

Based on the results of Sect. 2.3.2, which demonstrated loss of sustained growth under strictly bicarbonate-free conditions, a follow-up experiment was conducted to determine the minimal bicarbonate concentration required to restore reproducible metabolic activity under CO_2_-independent conditions. The objective was to assess whether bicarbonate fulfills a physiological role beyond extracellular pH stabilization and to quantify the concentration threshold necessary for stable growth. All experiments were performed under a 100% N_2_ headspace to exclude reintroduction of CO_2_-dependent carbonate equilibria. The minimal medium described in Sect. 2.2 was prepared without NaHCO_3_ and without the addition of a dedicated buffer. The medium was adjusted to pH 6.9 at room temperature, dispensed into serum bottles, rendered anaerobic under a 100% N_2_ atmosphere using repeated evacuation and overpressure cycles, and subsequently autoclaved at 121 °C for 20 min. After cooling, sterile NaHCO_3_ stock solutions were added aseptically to obtain final concentrations of 0, 0.125, 0.25, 0.375, 0.5, 1.0, 2.0, and 3.0 g L^− 1^ based on preliminary experiments (data not shown). The medium was not readjusted after NaHCO_3_ addition, and the pH immediately before inoculation was not determined separately for each bicarbonate concentration. To discriminate between bicarbonate-specific effects and potential sodium-associated influences, control conditions were established in which NaCl was added at a concentration equivalent to the molar Na⁺ contribution of the standard NaHCO_3_ treatment with 6 g/L. Serum bottles were inoculated with actively growing pre-cultures and incubated at 39 °C for seven days under otherwise identical conditions. Restoration of sustained growth was assessed by headspace pressure development, hydrogen production quantified by µGC, liquid metabolite formation measured by HPLC, and microscopic verification of rhizomycelial network formation. To minimize confounding effects from bicarbonate carry-over via the inoculum, cultures were propagated for at least two consecutive transfer cycles under the respective bicarbonate concentrations. Only growth and metabolic activity observed from the second transfer onward were considered indicative of stable bicarbonate-supported growth. All filamentous anaerobic fungi strains (*A. dupliciliberans*,* O. joyonii*,* P. ruminantium*, and *N. cameroonii*) were examined in biological triplicate.

#### Screening of alternative carboxylic acids

To determine whether bicarbonate can be functionally replaced by structurally distinct carboxylic acids, screening experiments were performed under a defined 100% N_2_ atmosphere. For the substitution experiment, all candidate compounds were evaluated individually in a bicarbonate-free basal medium. An otherwise identical medium supplemented with 4 g L^− 1^ NaHCO_3_ served as the positive control. The concentration of 4 g L^− 1^ NaHCO_3_ was selected to ensure non-limiting bicarbonate availability and therefore provided a suitable reference for comparison. Accordingly, each candidate compound was tested individually at a final concentration of 4 g L^− 1^. The screening was conducted exclusively with *A. dupliciliberans* A252 in biological triplicate. This strain was selected based on preliminary experiments demonstrating high hydrogen production and total metabolite formation under standard cultivation conditions, thereby providing a sensitive model for detecting growth-supporting or inhibitory effects of alternative carboxylates. The complete list of tested compounds is provided in Table S2. Each candidate acid was added individually to the bicarbonate-free base medium described in Sect. 2.2 at a final concentration of 4 g L^− 1^. Following addition, the medium pH was readjusted to 6.9 at room temperature prior to the establishment of anaerobic conditions to ensure comparability between treatments. A NaHCO_3_-containing medium (4 g L^− 1^, added after autoclaving) cultivated under identical N_2_ conditions served as positive control. All bottles were made anaerobic with 100% N_2_ using repeated evacuation and overpressure cycles, sterilized (121 °C, 20 min), supplemented with vitamin and antibiotic solutions, and inoculated with actively growing pre-cultures. Cultures were incubated at 39 °C for seven days. Following cultivation, growth performance was assessed by headspace pressure development, gas composition analysis (H_2_, CO_2_, N_2_) via µGC, and liquid-phase metabolite quantification by HPLC.

#### Metabolic screening using a dual buffer system

To systematically evaluate pH-dependent growth effects while minimizing carbonate-driven artefacts, a defined pH gradient experiment was performed under a 100% N_2_ atmosphere using a dual-buffer strategy. All cultures were established under nitrogen headspace conditions to eliminate CO_2_-dependent carbonate dynamics. The primary buffering system consisted of Bis-Tris (6 g L^− 1^), providing CO_2_-independent pH stabilization. Based on the bicarbonate titration experiment (Sect. 2.3.3), NaHCO_3_ was supplemented at a fixed concentration of 0.5 g L^− 1^, representing the lowest concentration previously shown to support reproducible growth. This concentration was selected to provide minimal inorganic carbon supplementation while limiting perturbation of the predefined pH gradient. Prior to making the medium anaerobic, the medium was adjusted at room temperature to defined initial pH values of 5.8, 6.1, 6.4, 6.7, or 7.0. Following nitrogen-based establishment of anaerobic conditions, serum bottles were sterilized (121 °C, 20 min). After cooling, NaHCO_3_, vitamin solution, and antibiotic solution were added aseptically. Cultures were inoculated with actively growing pre-cultures and incubated at 39 °C for seven days under otherwise identical conditions. All filamentous anaerobic fungi strains (*A. dupliciliberans* A252, *O. joyonii* W212, *P. ruminantium* SA222, *and N. cameroonii* G341) were tested in biological triplicate. At the end of incubation, metabolic activity was assessed by headspace pressure development, gas composition analysis (H_2_, CO_2_, N_2_) via µGC, and liquid-phase metabolite quantification by HPLC.

#### Establishment of biomass detection using OD_750_ and activated carbon

An OD_750_-based biomass calibration was established and validated using fungal dry biomass and activated carbon as an optical transfer reference. The complete calibration procedure, mathematical framework, and inter-device validation are provided in the Supplementary Information.

#### Impact of nitrogen source availability

To evaluate the influence of nitrogen source availability on fungal growth and metabolic performance, the nitrogen concentration of the cultivation medium was systematically varied while maintaining a constant carbon supply. The experiment was performed using *Caecomyces churrovis* PP313. This bulbous strain was selected due to its compact thallus morphology and reduced aggregation compared to extensively rhizoidal filamentous taxa, resulting in more homogeneous suspended biomass and improved suitability for turbidity-based quantification. Glucose was used as a defined and soluble carbon source at a fixed final concentration of 5 g L^− 1^ in all experiments. Nitrogen availability was modulated by varying the concentration of ammonium sulfate ((NH₄)_2_SO₄), the primary nitrogen source in the minimal medium. The following concentrations were tested: 0.3, 0.5, 1, 2, and 4 g L^− 1^. At constant glucose concentration (5 g L^− 1^), ammonium sulfate supplementation corresponded to nominal molar C/N ratios of 36.7, 22.0, 11.0, 5.5, and 2.75. These values refer to the variable ammonium sulfate fraction only. All cultures were established in 100 mL serum bottles containing 50 mL medium under standard CO_2_ atmosphere with 6 g L^− 1^ NaHCO_3_ buffering at an initial pH of 6.9, consistent with the conventional cultivation setup described in Sect. 2.2. After the establishment of anaerobic conditions and sterilization (121 °C, 20 min), bottles were inoculated with 5 mL actively growing pre-cultures and incubated at 39 °C for seven days. All conditions were performed in biological triplicate. At the end of the cultivation period, metabolic activity was assessed by headspace pressure measurement and gas composition analysis (µGC), while liquid-phase metabolites were quantified by HPLC. Biomass formation was determined using the OD_750_-based calibration framework described in Sect. 2.3.6.

### Analytics

#### Pressure measurement as indicator of metabolic activity

Headspace overpressure was determined after seven days of cultivation immediately prior to gas sampling and used as an indicator of metabolic activity. Detailed information on the measurement setup and instrumentation is provided in the Supplementary Information.

#### Gas chromatography for gas phase analysis

Headspace gas composition (H_2_, CO_2_, N_2_, and O_2_) was determined by micro gas chromatography (µGC). Detailed instrument configuration, operating conditions, and calibration procedures are described in the Supplementary Information.

#### High-performance liquid chromatography for liquid metabolite analysis

Liquid-phase metabolites were quantified by HPLC using external calibration with analytical standards. Details of sample preparation, chromatographic conditions, and calibration procedures are provided in the Supplementary Information.

#### Statistical analysis

Unless otherwise stated, cultivation experiments were performed in biological triplicate. For the comparison of CO_2_- and N_2_-based headspaces, biological triplicates were conducted across three independent weekly batches, resulting in *n* = 9 per strain and condition. OD_750_ calibration measurements were performed in ten technical replicates per concentration and photometric device. Quantitative data were summarized as arithmetic means ± standard deviation (SD), and error bars represent SD. Technical replicates were used to assess measurement variability and were not treated as independent biological replicates. Data were evaluated descriptively on the basis of headspace pressure, gas composition, liquid metabolite concentrations, OD_750_ values, and derived biomass estimates. For inter-device transfer of OD_750_ measurements, mean fungal OD_750_ values were transformed separately at each matched concentration using the ratio between the activated carbon responses of Photometer 2 and Photometer 1, as described in Sect. 2.3.6. Third-order polynomial fits were used only for graphical presentation and were not used to calculate the transferred OD_750_ values.

## Results and discussion

### Experimental overview

**Fig. 1:**
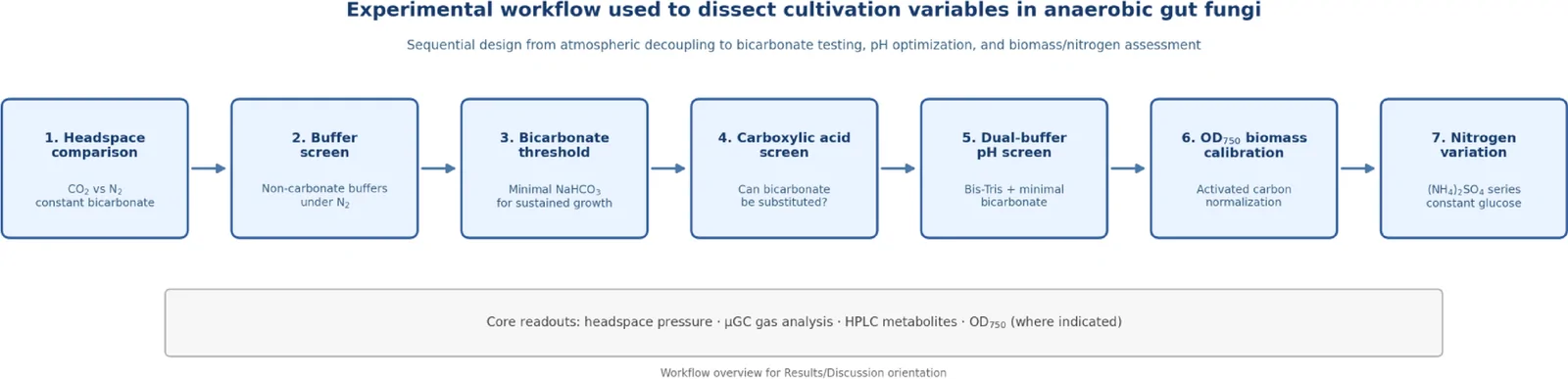
Experimental workflow used to systematically disentangle cultivation variables in anaerobic gut fungi (AGF). Starting from conventional CO_2_/HCO_3_⁻-based cultivation, the study sequentially evaluated (i) headspace composition (CO_2_ vs. N_2_), (ii) non-carbonate buffer systems, (iii) minimal bicarbonate requirements, (iv) substitution by structurally diverse carboxylic acids, (v) pH optima using a dual-buffer strategy, and (vi) an OD_750_-based biomass proxy, which was subsequently applied in an initial (vii) nitrogen-variation experiment. Core readouts included headspace pressure, µGC gas analysis, HPLC metabolite profiling, and OD_750_ where indicated

To guide interpretation of the results, the study was designed as a stepwise dissection of cultivation variables that are coupled in conventional AGF serum-bottle systems. CO_2_- and N_2_-based headspaces were first compared while retaining bicarbonate to isolate atmospheric effects, and non-carbonate buffers were then screened under N_2_ to test whether stable external pH can sustain growth in the absence of bicarbonate. Bicarbonate was subsequently reintroduced in defined concentrations and substituted by structurally diverse carboxylic acids to distinguish a quantitative inorganic carbon requirement from a more general carboxylate effect. Based on these findings, pH optima were determined using a dual-buffer strategy, after which an OD_750_-based biomass proxy was established and applied in an initial nitrogen-variation experiment (Fig. 1).

In the following analyses, total metabolite formation refers to the sum in mmol/L of quantified fermentation products measured in the liquid phase by HPLC (acetate, formate, lactate, succinate, malate, and citrate) and in the gas phase by µGC (hydrogen). The combined accumulation of these products is used as an integrative indicator of metabolic activity, as cumulative fermentation product formation correlates with active growth in anaerobic gut fungi [22].

### Headspace gas composition shifts hydrogen production

**Fig. 2:**
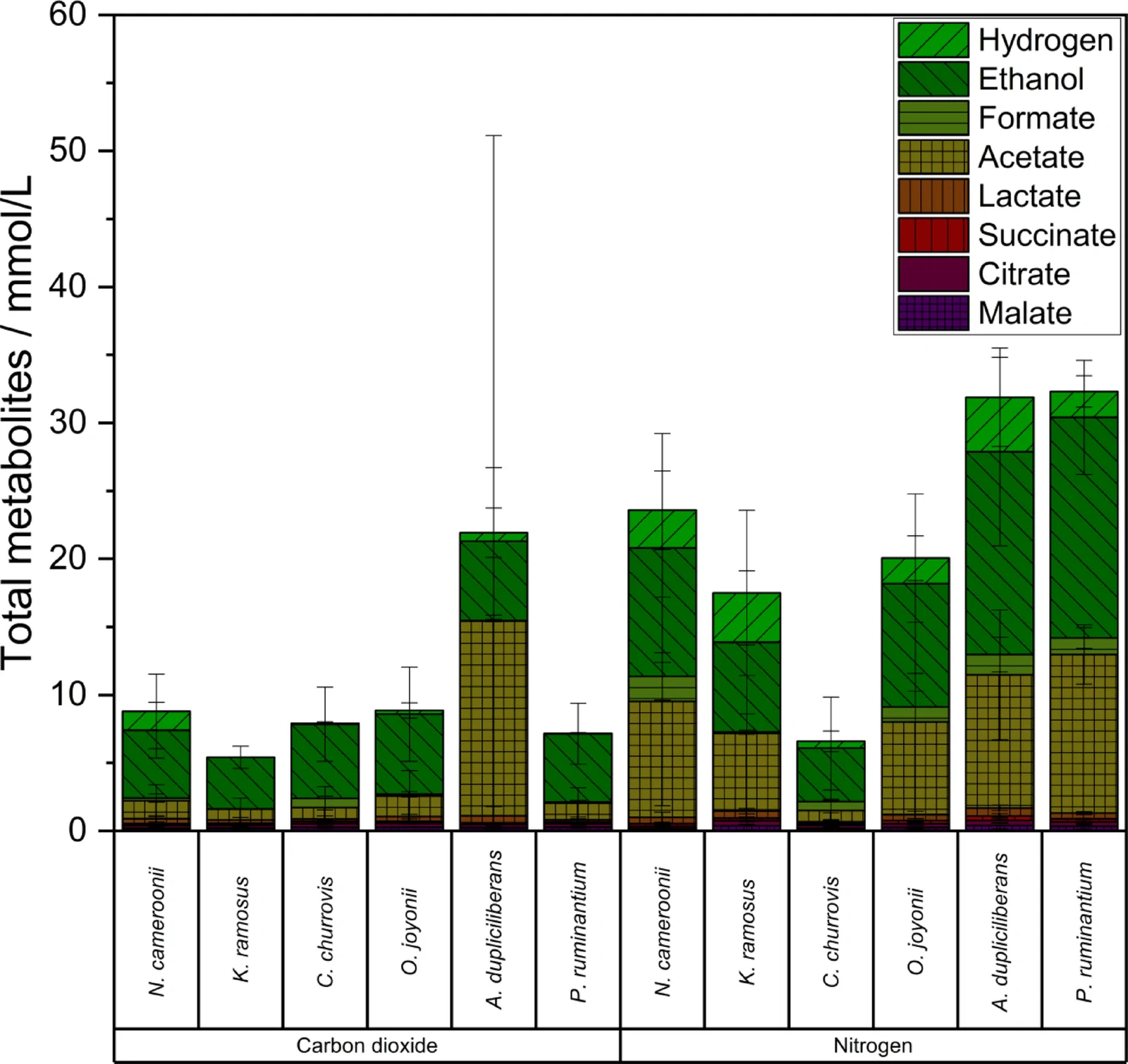
Atmospheric screening: influence of headspace gas composition (CO_2_ vs. N_2_) on metabolite production of six anaerobic gut fungal strains after 7 days of cultivation. All cultures were grown in identical bicarbonate-buffered medium and initiated at the same pH (7.5) to control for pH shifts associated with CO_2_ removal under N_2_. Cultivations were performed in biological triplicates across three independent weekly batches (*n* = 9). Error bars represent standard deviation. Hydrogen production was consistently higher under N_2_ headspace across all strains

To systematically dissect the physicochemical constraints governing anaerobic gut fungi cultivation, the influence of atmospheric composition was first evaluated while keeping the culture medium composition and pH constant. Because CO_2_, bicarbonate buffering, and dissolved-gas equilibria are tightly coupled in conventional cultivation systems, headspace composition is expected to affect redox balance, gas solubility, and metabolic flux distribution. To determine headspace effects independently from buffering chemistry, cultures were established under either CO_2_- or N_2_-based atmospheres while retaining identical medium composition and bicarbonate concentration. By adjusting both systems to the same initial pH, differences in growth performance and metabolite formation could be attributed specifically to atmospheric conditions rather than to pH variation or buffer substitution. Therefore, hydrogen production was quantified under CO_2_- and N_2_-based headspace conditions while maintaining identical bicarbonate-buffered medium and matched initial pH (Fig. 2).

Across all six strains, hydrogen formation was consistently elevated under an N_2_ atmosphere compared to CO_2_. In addition, total metabolite output was also higher under N_2_ in all strains except *C. churrovis*. This effect was particularly pronounced in strain *A. dupliciliberans*, which showed the highest hydrogen yield under N_2_ atmosphere. Hydrogen production in anaerobic gut fungi is associated with hydrogenosomal metabolism, where substrate oxidation generates reducing equivalents that are transferred to hydrogenases, resulting in molecular hydrogen formation [8]. Because hydrogen is a fermentation end product, its accumulation or removal from the system influences the thermodynamic feasibility of upstream redox reactions. Elevated hydrogen partial pressures are known to constrain fermentation by shifting reaction equilibria toward reduced intermediates, thereby limiting further substrate oxidation [24, 25]. However, H_2_-dependent thermodynamic constraints may not be the only mechanism contributing to the lower hydrogen production observed under a CO_2_-rich headspace. CO_2_ is itself a product of important reductant-generating hydrogenosomal reactions. Malic enzyme catalyzes the oxidative decarboxylation of malate to pyruvate and CO_2_ while reducing NAD(P)⁺, whereas pyruvate: ferredoxin oxidoreductase converts pyruvate and CoA to acetyl-CoA and CO_2_ while reducing ferredoxin [8, 26]. These reducing equivalents can subsequently provide electrons for hydrogen formation.

Because both enzyme classes are capable of catalyzing reverse reactions under appropriate biochemical conditions, the elevated dissolved CO_2_ concentration expected under a 100% CO_2_ headspace could, through a mass-action effect, oppose the oxidative, reductant-generating direction of these reactions [27, 28]. This could reduce the supply of NAD(P)H and reduced ferredoxin to hydrogenases independently of the H_2_ concentration in the liquid or gas phase. Consistent with a direct CO_2_-dependent effect on this pathway, pyruvate: ferredoxin oxidoreductase synthesis was previously reported to be suppressed when *N. patriciarum* was cultivated under a CO_2_ atmosphere [26]. The reduced hydrogen formation observed under CO_2_ may therefore reflect a combination of direct CO_2_-dependent constraints on upstream reductant-generating reactions and H_2_-dependent redox limitations. The latter may be particularly pronounced in axenic batch culture, where no hydrogen-consuming partner is present to relieve reducing pressure. In mixed or co-culture systems, methanogens can lower hydrogen partial pressure, stimulate cellulolytic enzyme activity, and increase hydrogenosomal metabolism, suggesting that the atmospheric response observed here reflects the unbuffered redox constraints of monoculture rather than the full in vivo rumen situation [29–31]. It is known that carbonic anhydrases link environmental CO_2_ availability to intracellular HCO_3_⁻ formation and downstream signaling/metabolism in aerobic fungi. Although this has not yet been demonstrated directly in AGF, it provides an additional plausible mechanism by which headspace composition could affect physiology even when bulk medium composition is unchanged [32–35]. Sensitivity of ruminal fungi to headspace composition has previously been reported, where altered gas atmospheres affected end-product patterns and fermentation intensity [10]. Recent work on *N. cameroonii* has demonstrated that hydrogen yield can be modulated by cultivation conditions, reflecting flexible metabolic flux distribution [25]. The present results extend these findings by showing that atmospheric composition alone, even in the presence of identical bicarbonate concentrations, consistently alters hydrogen output across multiple phylogenetically distinct strains. Although the initial inorganic carbon pool is distributed between the headspace and the liquid phase, this distribution changes continuously during cultivation due to fungal CO_2_ production and the dynamic CO_2_/HCO_3_⁻ equilibrium. Consequently, the biologically relevant availability of inorganic carbon cannot be inferred reliably from the initial distribution alone and was not quantified in the present study.

### Non-carbonate buffers stabilize pH but do not sustain bicarbonate-free growth

The six strains investigated represent both filamentous and bulbous AGF morphotypes. In the initial atmosphere screening (Fig. 2), filamentous strains showed higher and more reproducible hydrogen production than bulbous or bulbous-like strains. They were therefore prioritized for subsequent mechanistic experiments that required serial transfer and sustained growth under carbonate-independent conditions. The next step was to examine whether fungal growth can be sustained under strictly CO_2_-independent conditions when bicarbonate is completely removed from the medium. For this purpose, the bicarbonate buffer system was systematically replaced by chemically defined non-carbonate buffers and all cultivations were conducted under N_2_ atmosphere. This design enabled evaluation of buffering capacity independent of carbonate equilibria and assessment of whether alternative buffer systems can support growth in the absence of bicarbonate.

Because autoclaving and N_2_ treatment can substantially affect buffer performance, all candidate buffer systems were first evaluated for physicochemical stability following medium preparation. Only buffer–concentration combinations maintaining a post-sterilization pH of 6.9 ± 0.1 were selected for subsequent biological experiments. The complete buffer validation is provided in the Supplementary Information (Figure S3). For the subsequent biological evaluation of these pH-stable buffer systems, *N. cameroonii* G341 was selected as model strain because of its robust growth and consistent hydrogen production observed during the atmospheric screening.

**Fig. 3:**
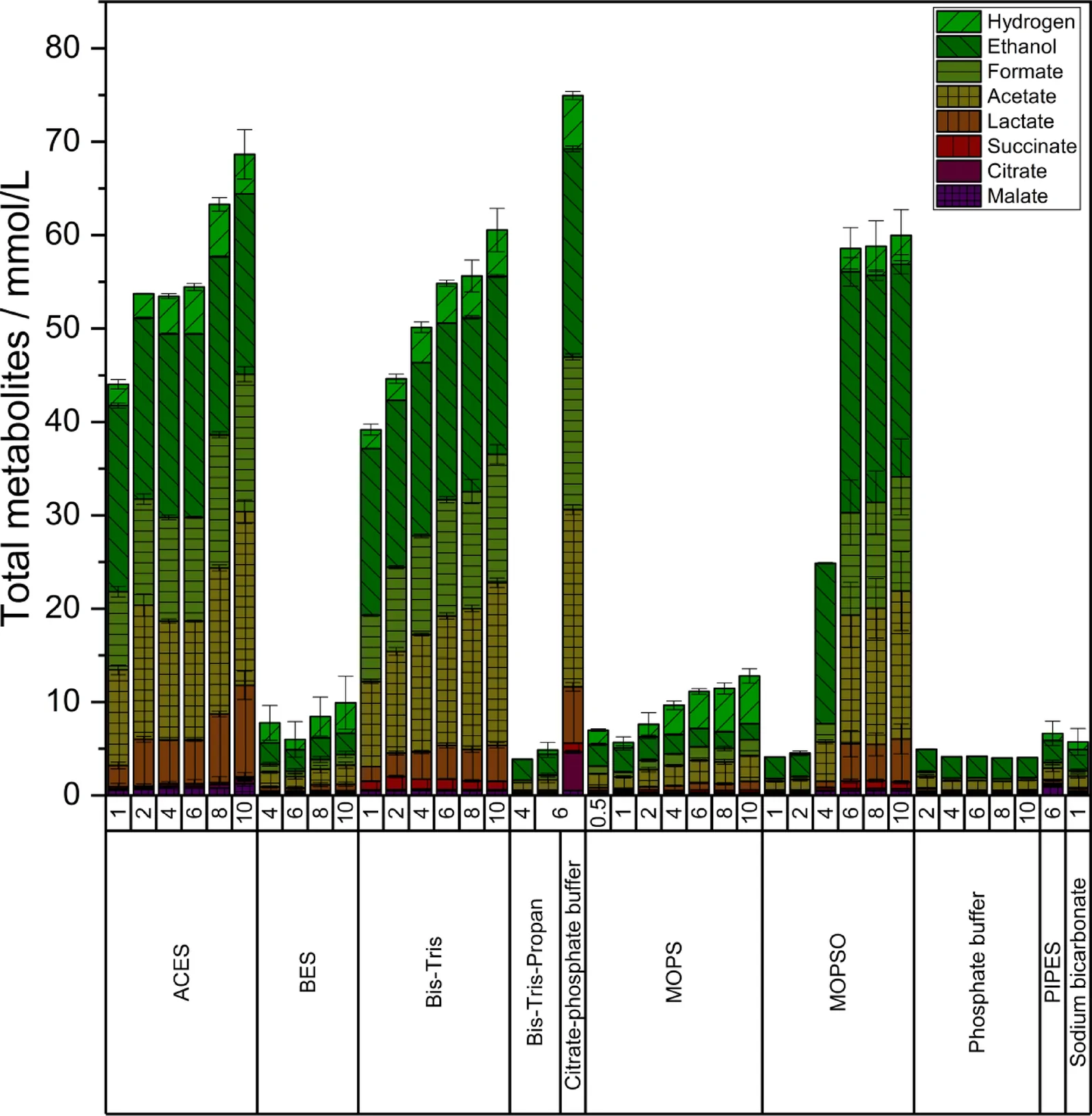
Total metabolite production (mmol per bottle) of N. cameroonii cultivated with chemically stable, bicarbonate-free buffer systems. Minimal medium was supplemented with the indicated concentration of the buffer systems, anaerobic conditions with N_2_ were established and the bottles were autoclaved. The 1 gl^-1^ sodium bicarbonate medium was prepared in the same order; therefore, this is not a positive control as it is lacking the CO_2_-gas phase that is normally part of this buffer system. Only buffer–concentration combinations maintaining post-sterilization pH 6.9 ± 0.1 were evaluated. Limited metabolic activity was observed during the first cultivation cycle (shown above); however, no sustained hydrogen production or metabolite formation was detected after transfer into fresh medium in the subsequent week. Data represent biological triplicates (*n* = 3); error bars indicate standard deviation. For ACES-containing samples, apparent succinate signals reflect chromatographic co-elution; a representative fixed background value (0.2 mmol L^− 1^) is shown. The numbers on the X-Axis indicate the concentration of the buffer in gl^-1^

Subsequently, only those buffer–concentration combinations that maintained the target pH (6.9 ± 0.1) after the establishment of anaerobic conditions and autoclaving were selected for biological evaluation under N_2_ atmosphere (Table S1). Because inoculation was performed from standard NaHCO_3_/CO_2_ precultures, residual dissolved inorganic carbon could not be fully excluded during the first transfer cycle. Figure 3 therefore summarizes the first-transfer growth performance of the model organism *N. cameroonii* in these pH-stable buffer systems. It should be noted that the NaHCO_3_ reference medium included in this experiment was prepared by adding bicarbonate before N_2_ anaerobization and autoclaving. Consequently, removal of CO_2_ during N_2_ treatment shifted the carbonate equilibrium and substantially reduced the remaining dissolved inorganic carbon pool, although the post-sterilization pH remained within the predefined acceptance range. Thus, this condition represents a pH-stable bicarbonate-buffered reference after N_2_ treatment rather than a positive control with a defined bicarbonate concentration. While limited metabolic activity was still detectable in the initial transfer culture, no measurable growth, hydrogen production, or sustained metabolite formation was observed after subsequent transfers, when inoculum-associated carbonate carry-over was no longer expected to contribute appreciably. These results indicate that stable external pH alone is insufficient to sustain growth in the absence of bicarbonate. One plausible explanation is that, once inoculum-associated carryover is depleted, intracellular bicarbonate supply becomes limiting for carbonic-anhydrase-mediated CO_2_ hydration and downstream bicarbonate-dependent metabolic or signaling processes. In fungi, carbonic anhydrases are broadly implicated in maintaining bicarbonate homeostasis and coupling environmental CO_2_ availability to cellular physiology [32, 33].The failure to re-establish growth after transfer argues against a purely transient adaptation effect and supports the interpretation of a physiological requirement for carbonate not explained by extracellular pH control alone. Because AGF energy metabolism depends on hydrogenosomal pyruvate oxidation and associated redox balancing [8], removal of bicarbonate may disturb central metabolic fluxes rather than merely extracellular pH. This interpretation is consistent with bicarbonate-dependent carboxylation reactions described in other anaerobic systems [19].

**Fig. 4:**
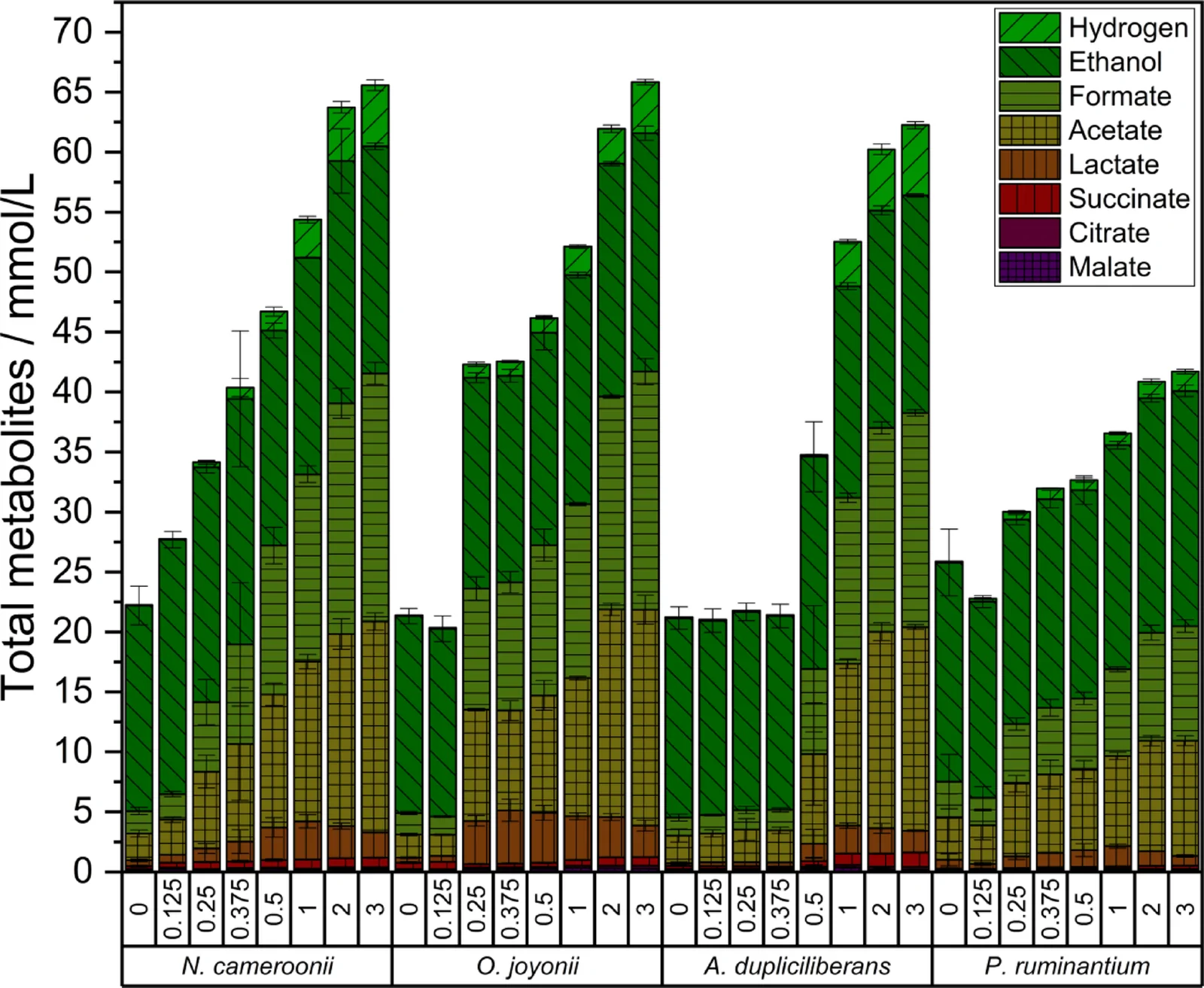
Strain-specific restoration of metabolic activity by sodium hydrogen carbonate under a 100% N_2_ atmosphere. Total metabolite production (mmol L^− 1^) of filamentous anaerobic gut fungal strains (A. dupliciliberans, N. cameroonii, O. joyonii, and P. ruminantium) is shown for the second cultivation cycle across increasing sodium hydrogen carbonate concentrations (0–3 g L^− 1^). Carbonate-free conditions (0 g L^− 1^ NaHCO_3_) resulted in negligible hydrogen formation, whereas sodium hydrogen carbonate supplementation restored hydrogen production in a strain-specific, concentration-dependent manner. Data represent biological triplicates (*n* = 3); error bars indicate standard deviation. The numbers on the X-Axis indicate the concentration of NaHCO_3_ in g L^-1^

Because the primary objective of this experiment was to identify chemically stable non-carbonate buffer systems, conclusions from Fig. 3 should not be drawn from the NaHCO_3_ reference itself. Instead, the key observation is that none of the bicarbonate-free buffer systems supported sustained growth after serial transfer despite maintaining stable external pH. The physiological requirement for bicarbonate was subsequently investigated under defined bicarbonate concentrations in the dedicated concentration-response experiment shown in Fig. 4, in which NaHCO_3_ was added only after N_2_ treatment and sterilization.

### Low bicarbonate thresholds restore activity under N_2_

To further examine the physiological function of bicarbonate, sodium hydrogen carbonate was systematically reintroduced into carbonate-free medium under nitrogen atmosphere across a defined concentration gradient (0, 0.125, 0.25, 0.375, 0.5, 1, 2, and 3 g L^− 1^). Figure 4 summarizes the strain-specific response of the filamentous anaerobic gut fungi *A. dupliciliberans*, *N. cameroonii*, *O. joyonii*, and *P. ruminantium* to increasing bicarbonate availability. In the absence of bicarbonate (0 g L^− 1^), hydrogen production and total metabolite formation were negligible across all tested strains, consistent with the absence of sustained growth under carbonate-free conditions. Upon gradual supplementation of sodium hydrogen carbonate, hydrogen formation reappeared in a concentration-dependent manner. For *N. cameroonii* and *O. joyonii*, reproducible metabolic activity was restored at low bicarbonate concentrations (≥ 0.25 g L^− 1^), whereas *A. dupliciliberans* required slightly higher levels (≥ 0.5 g L^− 1^) to re-establish stable hydrogen production. *P. ruminantium* exhibited a similar recovery pattern but with reduced overall hydrogen output compared to *N. cameroonii*. Notably, hydrogen production did not increase proportionally with bicarbonate concentration beyond the minimal threshold; instead, a plateau-like behavior was observed once sufficient bicarbonate was available. For better visualization hydrogen production of *N. cameroonii* is shown exclusively in Figure S5. This pattern is consistent with a threshold-dependent requirement rather than a simple concentration-dependent stimulation effect. The concentration-dependent recovery of hydrogen formation further supports a physiological role of bicarbonate in anaerobic gut fungal metabolism. Because NaHCO_3_ was added after the establishment of anaerobic conditions and sterilization and the pH was not remeasured after supplementation, a concentration-dependent pH shift cannot be excluded. However, the pronounced reduction in metabolic activity occurred toward the lower end of the bicarbonate concentration range, whereas no decline was observed at the highest concentrations tested. An inhibitory alkaline shift therefore does not appear to be the primary cause of the observed plateau, although a contributing effect of pH cannot be ruled out. The threshold-like character of this response is compatible with a system in which a minimum external bicarbonate level is required before intracellular HCO_3_⁻ pools become sufficient to support bicarbonate-dependent carboxylation and related metabolic functions, rather than with a simple linear stimulation by increasing buffer concentration [18, 19, 32]. The strain-specific bicarbonate thresholds observed may be explained with the broader physiological diversity reported across AGF taxa and may reflect differences in intracellular inorganic carbon handling, hydrogenosomal flux partitioning, or carboxylation capacity rather than a single universal bicarbonate requirement [1, 36]. Early physiological studies on *Neocallimastix* demonstrated that carbon dioxide availability affects fungal growth and metabolism [21]. Furthermore, genomic analyses of anaerobic gut fungi have identified carboxylation-associated enzymes that may require CO_2_-derived intermediates in central metabolism [36]. To assess whether the sodium contribution of the standard bicarbonate-containing medium accounted for the observed growth-supporting effect, a separate control containing NaCl was tested. The used concentration was equimolar with respect to the Na⁺ supplied by 6 g L^− 1^ NaHCO_3_. As shown in Figure S4, NaCl supplementation did not restore hydrogen production or sustained metabolite formation, indicating that sodium ions alone were insufficient to reproduce the effect of bicarbonate.

### Alternative carboxylates do not substitute bicarbonate

**Fig. 5:**
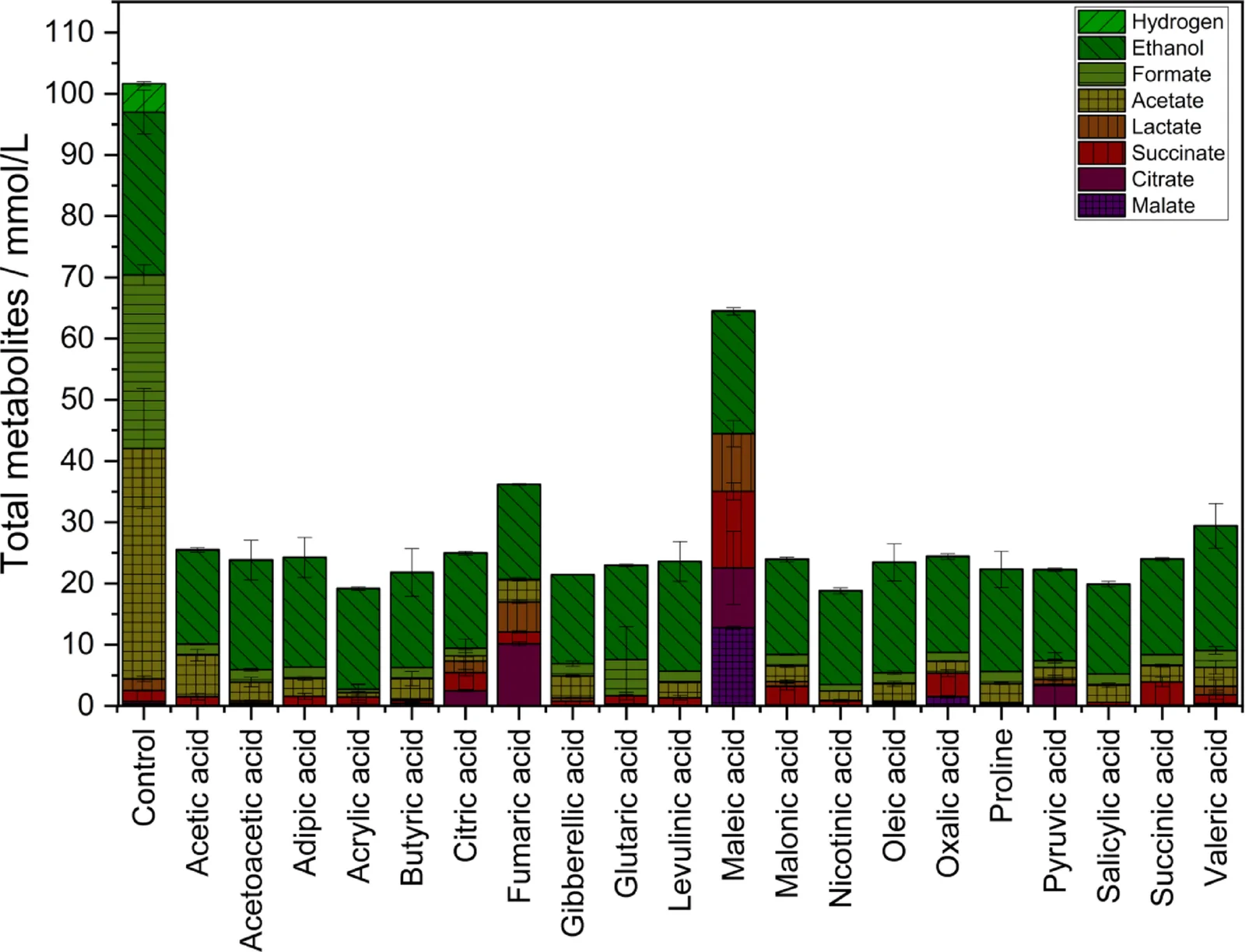
Metabolite production by A. dupliciliberans under a 100% N_2_ atmosphere in media with structurally diverse carboxylic acids. Stacked bars show the individual and total concentrations of quantified metabolites (hydrogen, ethanol, formate, acetate, lactate, succinate, citrate, and malate) after seven days of cultivation in bicarbonate-free medium supplemented with 4 g L^− 1^ of the indicated carboxylic acid; the control contained 4 g L^− 1^ sodium hydrogen carbonate. Values were corrected for compound-specific background and co-elution artefacts identified by independent standard measurements. None of the tested carboxylic acids restored the bicarbonate-supported hydrogen production observed in the control. Maleic acid yielded the highest metabolite accumulation among the tested carboxylic acids; however, acetate is not shown for this condition because maleic acid can isomerize and generate an interfering HPLC signal, preventing reliable acetate quantification. Data represent biological triplicates (*n* = 3); error bars indicate standard deviation

The concentration-dependent restoration of metabolic activity by sodium hydrogen carbonate (Fig. 4) is consistent with a bicarbonate requirement for sustained growth under nitrogen atmosphere. However, this finding does not resolve whether the observed dependency is specific to bicarbonate itself or whether other structurally related carboxylate-containing compounds could functionally substitute its role. To address this question, alternative carboxylic acids were systematically screened under N_2_ atmosphere. This screening was conducted exclusively with *A. dupliciliberans*, selected based on its high hydrogen production and total metabolite formation in preceding experiments. The use of a metabolically robust strain increases sensitivity for detecting both growth-supporting and inhibitory effects of alternative carboxylates. The results of this screening are summarized in Fig. 5. Across all tested carboxylic acids, total metabolite formation remained substantially lower than in the NaHCO_3_ control, and hydrogen production did not recover to bicarbonate-supported levels. This pattern indicates that none of the screened organic carboxylates was able to functionally substitute bicarbonate in restoring the characteristic hydrogenosomal fermentation phenotype. Instead, end-product profiles were dominated by metabolites typically associated with cytosolic redox balancing, including lactate, succinate, and malate, whereas hydrogen formation remained comparatively low. In anaerobic metabolism more broadly, dissolved inorganic carbon participates in anaplerotic C_3_-carboxylation reactions that replenish oxaloacetate pools and sustain central carbon flux. Limitation of intracellular CO_2_/HCO_3_⁻ availability can constrain anaplerosis and growth, whereas supplementation can restore metabolic flux distribution [18, 19]. Consistent with fungal CO_2_/HCO_3_⁻ signaling models, carbonic anhydrase activity might generate bicarbonate that can be sensed directly or indirectly by cAMP-associated pathways. Under such a framework, externally supplied organic carboxylates would not be expected to substitute for bicarbonate if the critical requirement is intracellular HCO_3_⁻ generation rather than provision of a generic organic acid [32, 34, 35]. Together, these observations are consistent with the interpretation that bicarbonate contributes to central metabolic flux beyond extracellular pH control. An additional consideration is that several tested organic acids may impose weak-acid stress rather than simply acting as alternative carbon-bearing species. In fungi, membrane-permeant weak acids can cause cytosolic acidification, anion accumulation, and ATP-consuming pH homeostasis responses, which may suppress growth even when extracellular pH is readjusted before inoculation [37–39]. However, maleic acid represented a notable exception. Maleate supplementation resulted in elevated apparent total metabolite formation compared to other tested acids, yet this increase was not accompanied by proportional hydrogen production. Detected metabolites indicate a redistribution of carbon flux toward cytosolic end products rather than restoration of balanced hydrogenosomal metabolism. One possible explanation relates to the chemical properties of unsaturated dicarboxylates such as maleate and fumarate. In various anaerobic systems, fumarate can function as an electron acceptor and support cytosolic redox balancing through reduction to succinate [40, 41]. Provision of structurally related compounds may therefore influence intracellular redox distribution without replacing bicarbonate-dependent anaplerotic flux. In addition, maleate has been reported to interact with enzymes of central carbon metabolism, including fumarase and pyruvate kinase [42, 43], potentially contributing to altered carbon partitioning. These mechanisms remain speculative in the context of anaerobic gut fungi but may explain the observed decoupling between soluble metabolite formation and hydrogen production. Future experiments should further distinguish between buffering effects and inorganic carbon supply, for example by combining non-carbonate pH buffers with defined CO_2_ dosing or supplementation with specific C₄ intermediates to probe potential anaplerotic limitations. An additional compound of interest for future studies is formate, as its conversion to CO_2_ and H_2_ in many anaerobic microorganisms may provide further insight into whether the observed dependency primarily reflects inorganic carbon availability or a more specific physiological requirement for bicarbonate.

### Dual-buffer cultivation identifies a pH optimum at 6.4

**Fig. 6:**
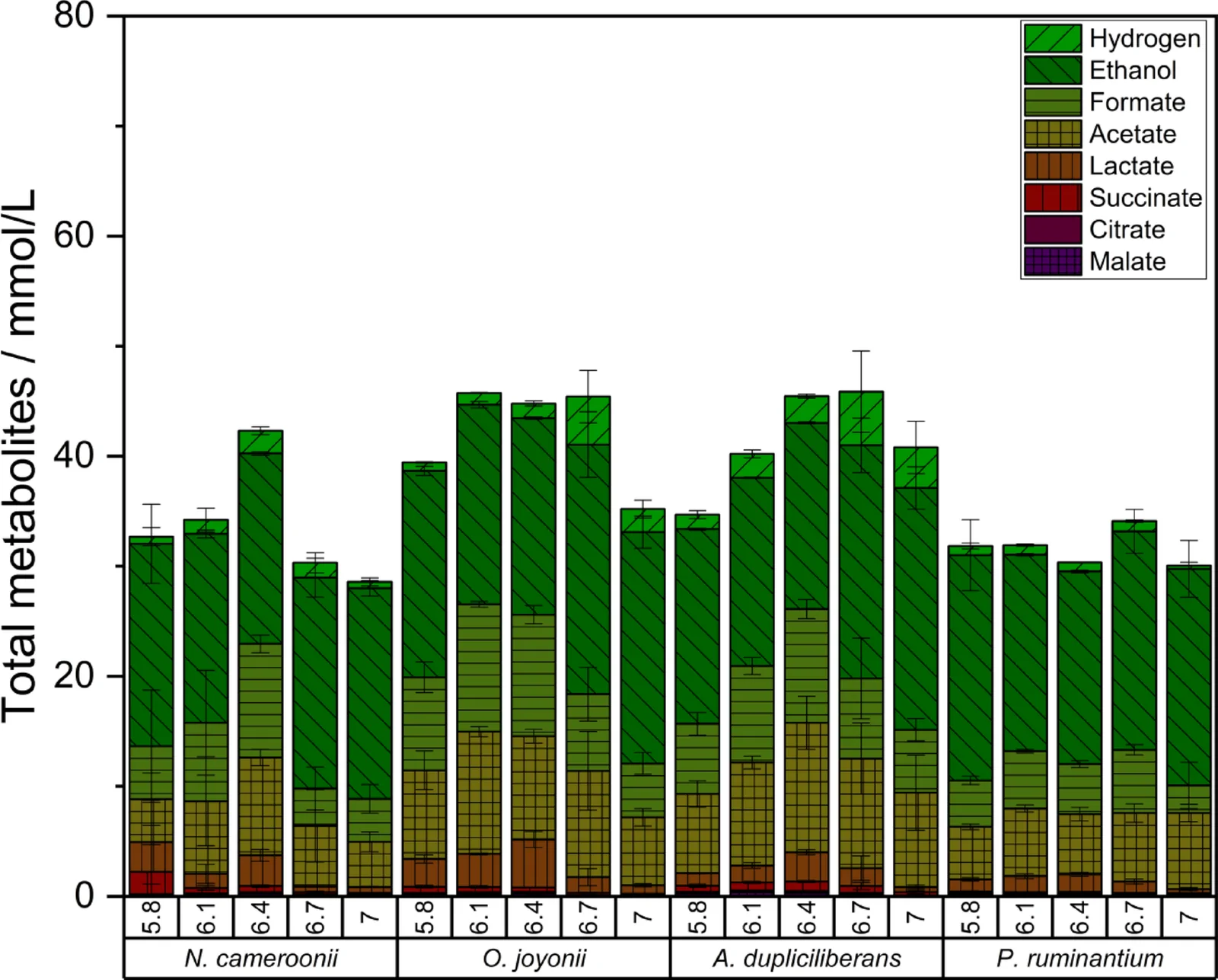
Total metabolite production by four filamentous anaerobic gut fungal strains under a 100% N2 atmosphere at initial pH between 5.8 and 7. Cultures were grown with Bis-Tris (6 g L^− 1^) as the primary buffer and 0.5 g L^− 1^ NaHCO_3_ as a minimal inorganic carbon supplement. Data represent biological triplicates (*n* = 3); error bars indicate standard deviation

To determine pH optima under nitrogen atmosphere, a dual-buffer strategy was implemented in which pH stabilization was provided by Bis-Tris Buffer while a minimal concentration of sodium hydrogen carbonate (0.5 g L^− 1^) was supplied as a defined inorganic carbon supplement. This experimental design allowed independent control of external pH while maintaining a constant, low bicarbonate background. Only filamentous anaerobic gut fungal strains were evaluated in this mechanistic assay (Fig. 6). Across the tested pH range (5.8–7.0), hydrogen production and total metabolite formation were generally highest around pH 6.4, although the strength of this response differed between strains. In contrast, *P. ruminantium* exhibited only minor differences in metabolic activity across the tested pH range, indicating a comparatively weak pH-dependent response under the applied cultivation conditions. At more acidic conditions (pH 5.8–6.1), metabolic output declined, whereas at neutral conditions approaching pH 7.0, hydrogen production was reduced relative to the optimum. The observation that pH 6.4 yields maximal activity is biologically consistent with the natural ruminal environment. In vivo measurements of rumen fermentation typically report a physiological pH range between approximately 6.0 and 6.8, depending on diet and fermentation intensity [44]. Anaerobic gut fungi are adapted to this mildly acidic niche and are known to exhibit optimal growth and fermentation activity within this window [21]. Deviations toward higher pH may reduce proton availability for redox reactions and alter membrane potential dynamics, whereas excessive acidity can impair enzyme activity and intracellular homeostasis. Importantly, because Bis-Tris buffer maintained stable external pH independent of CO_2_/HCO_3_⁻ equilibria, the observed optimum is consistent with a physiological pH preference rather than an artefact of carbonate buffering. It should be noted, however, that the relative buffering contributions of Bis-Tris and bicarbonate are expected to vary across the investigated pH range because of their overlapping pKa values. In addition, phosphate present in the basal medium may contribute to the overall buffering capacity, particularly at higher pH values. Consequently, the dual-buffer system should not be interpreted as consisting of completely independent buffering components. Nevertheless, because bicarbonate was supplied at a constant concentration throughout this experiment, the observed differences are considered to primarily reflect physiological responses to external pH rather than changes in inorganic carbon availability. This distinction may be particularly relevant for bicarbonate handling, because carbonic-anhydrase-catalyzed interconversion of CO_2_ and HCO_3_⁻ links inorganic carbon availability to proton balance and thereby integrates pH and carbon supply at the cellular level in fungi [32, 33]. Hydrogen production in anaerobic gut fungi is associated with hydrogenosomal pyruvate oxidation [8], and hydrogenosomal enzymes are sensitive to intracellular pH conditions. The alignment of maximal hydrogen production at pH 6.4 therefore supports the interpretation that hydrogenosomal metabolism is tuned to rumen-like acidity. Notably, even at the optimal pH, hydrogen production did not exceed levels observed under full bicarbonate supplementation in earlier experiments. Taken together, the results of the preceding experiments indicate that atmospheric composition, bicarbonate availability, and extracellular pH should not be considered as independent cultivation variables but as interacting components of a common physiological framework. Headspace composition determines the availability of gaseous CO_2_, bicarbonate provides dissolved inorganic carbon while simultaneously contributing to extracellular buffering, and pH influences both carbonate speciation and enzyme activity. The present data suggest that sustained hydrogenosomal metabolism requires not only a suitable extracellular pH but also sufficient inorganic carbon availability. Consequently, the reduced activity observed under bicarbonate-free conditions cannot be explained solely by impaired pH regulation, but is consistent with a physiological requirement for inorganic carbon to support balanced carbon metabolism. The dual-buffer approach presented here therefore provides a practical framework for experimentally separating these previously coupled variables and enables more mechanistic investigation of anaerobic gut fungal physiology. This also suggests that the identified pH optimum is conditional on the low-bicarbonate background used in the dual-buffer setup and may shift under conditions of higher inorganic carbon supply or continuous product removal. Continuous-culture studies have shown that AGF metabolic fluxes can be tuned by cultivation regime, indicating that pH optima should be interpreted in the context of both carbon availability and product accumulation [45]. This finding suggests that bicarbonate contributes metabolic functions beyond external pH stabilization, potentially through inorganic carbon availability required for balanced carbon flux and energy metabolism.

### OD_750_ calibration provides a practical biomass proxy

Because reliable biomass quantification remains challenging in anaerobic gut fungi, an OD_750_-based biomass proxy was established and validated for the cultivation conditions used in this study. The complete calibration procedure and inter-device validation are provided in the Supplementary Information (Figure S6). Overall, the calibration demonstrated that activated carbon can serve as a practical optical transfer reference, allowing approximate comparison of OD_750_ measurements between photometric systems. The resulting biomass proxy was subsequently applied in the nitrogen-variation experiment described below.

Having established N_2_ as an inert headspace gas for decoupling atmospheric composition from bicarbonate chemistry, we next considered nitrogen in a conceptually distinct role: as an assimilable nutrient within the cultivation medium. Molecular nitrogen in the headspace was not investigated as a nutritional nitrogen source. Rather, the following experiments represent an initial extension of the decoupled cultivation framework toward future medium optimization. For such studies, a biomass proxy is required to distinguish changes in metabolic output from changes in fungal biomass formation. An OD_750_-based calibration was therefore established and subsequently applied in a proof-of-principle experiment varying ammonium sulfate concentration

### Nitrogen variation has limited effects within the tested range

**Fig. 7:**
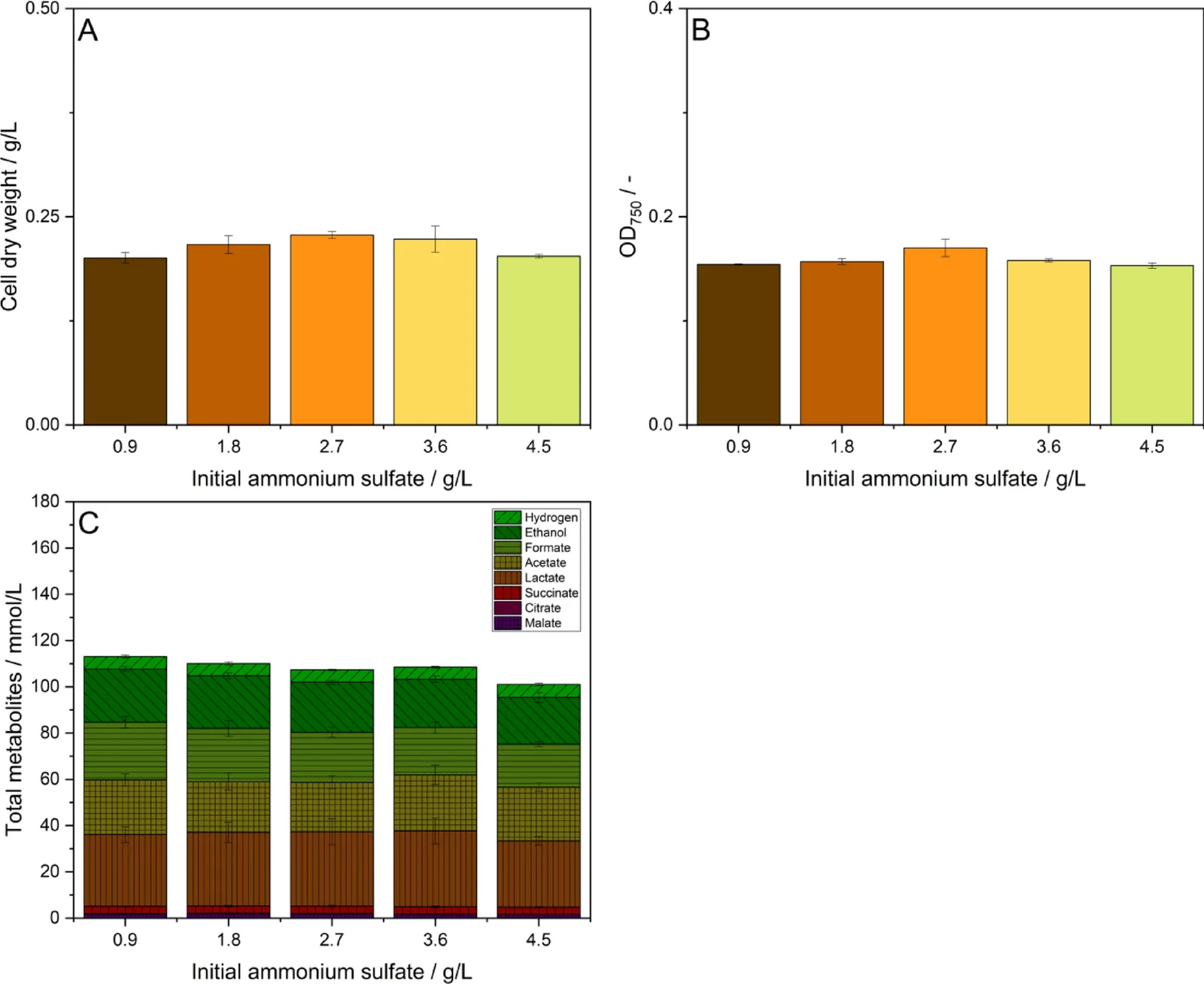
Impact of ammonium sulfate concentration on biomass formation and metabolic output. **A**: Biomass (OD_750_-derived dry mass); **B**: OD₇₅₀ values; **C**: Total metabolites (mmol L^-1^) of Caecomyces churrovis PP313 with varying ammonium sulfate concentrations. No major differences in biomass accumulation or hydrogen production were observed across the tested nitrogen range, indicating non-limiting nitrogen conditions. Data represent biological triplicates; error bars indicate standard deviation

While Figure S6 establishes a transferable OD_750_-based biomass detection framework, its practical applicability must be evaluated under defined physiological perturbations. To assess both the sensitivity of the method and the influence of nutrient balance on fungal growth, the impact of nitrogen availability on biomass formation and metabolic performance was investigated. *C. churrovis* was selected for this experiment due to its bulbous growth morphology characterized by compact thalli and reduced rhizoidal aggregation compared to filamentous taxa. This morphology results in more homogeneous suspension behavior in liquid culture and reduced clumping, thereby improving the reliability of turbidity-based biomass measurements. Because OD-based quantification relies on light scattering of suspended particles, a strain with minimal aggregation artifacts provides a suitable system for evaluating changes in optical density under variable nutrient conditions. The results are presented in Fig. 7. Across the tested ammonium sulfate concentrations, no consistent trend in biomass accumulation was observed. OD_750_ values and calculated biomass concentrations remained within a narrow range across all nitrogen levels and did not display a clear dose–response relationship. Similarly, total metabolite formation and hydrogen production remained comparable across the tested concentration range. These observations indicate that, within the applied concentration window, nitrogen availability did not impose a detectable growth limitation under the experimental conditions. At the same time, this conclusion applies specifically to the defined glucose-based, bicarbonate-buffered medium used here, which also contained constant background nitrogen from additional supplements. The absence of a response therefore likely indicates that the tested ammonium sulfate window remained above the threshold of true nitrogen limitation rather than that nitrogen is generally unimportant for AGF physiology. Comparable findings have been reported in other fungal systems, where growth reduction becomes apparent only when nitrogen availability drops below a threshold required to sustain anabolic processes [46]. The stability of hydrogen production across nitrogen concentrations further suggests that hydrogenosomal metabolism was not constrained by nitrogen supply within the tested range. Taken together, these results indicate that, under the present cultivation conditions, atmospheric composition and inorganic carbon availability exert stronger effects on metabolic output than moderate variations in nitrogen concentration. Future experiments extending into clearly nitrogen-limited regimes, combined with direct quantification of residual ammonium and cellular protein content, would be required to resolve potential C: N-dependent regulatory effects in anaerobic gut fungi.

## Conclusions

This study systematically disentangles atmospheric composition, buffer chemistry, bicarbonate availability, pH control, and ammonium concentration as interacting variables in anaerobic gut fungal growth and metabolism. The results show that conventional CO_2_-based cultivation intrinsically couples gas-phase composition with carbonate buffering, complicating mechanistic interpretation. By implementing nitrogen headspace conditions and non-carbonate buffer systems, atmospheric and buffering effects could be experimentally separated. The results are consistent with a physiological role of bicarbonate beyond extracellular pH stabilization. Complete removal of bicarbonate abolished sustained growth even under stable pH conditions, whereas reintroduction at low concentrations restored hydrogen production in a strain-specific manner. Alternative organic carboxylic acids did not substitute this function. Although maleic acid induced elevated cytosolic metabolite accumulation, this response was not accompanied by proportional hydrogen formation and did not restore the balanced hydrogenosomal fermentation phenotype observed under bicarbonate supplementation. The dual-buffer pH experiment identified maximal hydrogen production at pH 6.4 under nitrogen atmosphere, consistent with reported ruminal pH conditions. However, pH optimization alone did not compensate for bicarbonate limitation. Furthermore, variation of ammonium sulfate within the tested range did not impose a detectable growth limitation, indicating that nitrogen availability was not the dominant regulatory factor under the applied conditions. The establishment of a transferable strain specific OD₇₅₀-based biomass calibration framework enables reliable, non-invasive quantification of fungal growth across experimental perturbations and supports discrimination between altered metabolic flux and biomass effects. Collectively, these findings indicate that atmospheric composition and inorganic carbon availability are key determinants of metabolic performance in anaerobic gut fungi. Several targeted follow-up experiments are required to further resolve the precise physiological role of bicarbonate. Future studies should more rigorously decouple inorganic carbon supply from buffering effects, for example by combining non-carbonate pH buffers with controlled CO_2_ dosing under N_2_ atmospheres. Because the distribution of inorganic carbon between the gas and liquid phases changes continuously during cultivation due to fungal CO_2_ production and carbonate equilibration, future studies should additionally include time-resolved quantification of dissolved inorganic carbon to better resolve the relative contributions of headspace and dissolved inorganic carbon availability. Direct quantification of dissolved inorganic carbon, alongside supplementation with defined C₄ intermediates (e.g., malate, succinate, fumarate, or aspartate), would help clarify whether bicarbonate primarily supports anaplerotic carbon flux, intracellular redox balance, or both. Additional time-resolved and multi-strain analyses will be required to determine the generality of bicarbonate thresholds and pH optima across *Neocallimastigomycota*. Such mechanistic resolution is essential for developing reproducible and scalable cultivation strategies for the biotechnological exploitation of anaerobic gut fungi.

## Supplementary Information

Below is the link to the electronic supplementary material.

Supplementary Material 1.

## Data Availability

The datasets used and/or analyzed during the current study are available from the corresponding author on reasonable request.
