# Supplementary material for: Beyond carbon dioxide: decoupling headspace and buffering reveals a bicarbonate requirement for anaerobic gut fungi growth
Source: Fungal Biol Biotechnol. 2026 Oct 7;13:18. doi: 10.1186/s40694-026-00224-0 (PMC13644296; doi:10.1186/s40694-026-00224-0)
Supplement: Supplementary file 1 [file 40694_2026_224_MOESM1_ESM.pdf]

Supplementary Information to: "Beyond Carbon Dioxide: Decoupling Headspace and Buffering Reveals a Bicarbonate Requirement for Anaerobic Gut Fungi Growth"

Methods:

S 2.3.6 Establishment of Biomass detection Using OD_{750} and activated carbon

To establish a reproducible and transferable OD-based biomass quantification method for anaerobic gut fungi, a two-step calibration strategy was implemented. The approach was designed to address both the morphological complexity of AGF cultures and the need for inter-device comparability while minimizing repeated fungal biomass production for calibration purposes. Fungal biomass was generated in 2 L batch cultivations using glucose as sole carbon source (5 g L^{-1}) to ensure homogeneous growth without interference from particulate substrates. Cultures of all six strains were incubated under standard anaerobic conditions at 39 °C until sufficient biomass accumulation was achieved. The biomass was harvested by centrifugation, washed repeatedly with sterile 0.9 g L^{-1} saline solution to remove soluble metabolites, and subsequently freeze-dried to constant weight to obtain defined dry mass samples. Defined dry masses of fungal biomass were weighed and resuspended in saline solution. From these, twelve defined concentration levels were prepared (0.005–0.5 g L^{-1}). Optical density measurements were performed at 750 nm using two independent photometric systems (SPECTRONIC 200 and GENESYS™ 30, Thermo Scientific, USA). Each concentration was measured in ten technical replicates per device and mean values with standard deviations were calculated to assess intra-device variability and inter-device comparability. The wavelength of 750 nm was selected to ensure that measurements predominantly reflected light scattering (turbidity) rather than wavelength-dependent optical absorption. In parallel, activated carbon suspensions were prepared at the same twelve dry-mass concentrations and measured on both photometric systems under identical conditions. Each concentration was measured in ten technical replicates per device. Activated carbon was used as an optical transfer reference to account for differences in instrument response.

The transfer was calculated separately for each matched concentration rather than by applying a single correction factor across the entire concentration range. For fungal strain s at concentration c, a quotient was first calculated from the mean activated carbon and fungal OD_{750} values measured on Photometer 1:

$$q(s,c) = OD(AC,P1,c) \,/\, O\bar{D}(s,P1,c) \qquad (1)$$

where OD(AC,P1,c) denotes the mean activated carbon OD_{750} and OD(s,P1,c) the mean OD_{750} of fungal strain s at the same dry-mass concentration measured on Photometer 1. The expected fungal OD_{750} value on Photometer 2 was subsequently calculated using the activated carbon measurement obtained with Photometer 2:

$$OD(s,P2,c) = OD(AC,P2,c) / q(s,c) \qquad (2)$$

This calculation is algebraically equivalent to:

$$OD(s,P2,c) = OD(s,P1,c) \times [OD(AC,P2,c) / OD(AC,P1,c)] \qquad (3)$$

Thus, the ratio between the activated carbon responses of the two instruments was used as a concentration-specific inter-device transfer factor and applied to the fungal OD_{750} value measured on Photometer 1. Each calculated value was then compared with the corresponding fungal biomass suspension measured directly on Photometer 2.

The absolute percentage deviation between the calculated and directly measured OD_{750} values was determined as:

$$\text{Absolute percentage deviation} = |1 - [O\hat{D}(s,P2,c) / O\bar{D}(s,P2,c)]| \times 100 \qquad (4)$$

Third-order polynomial curves were fitted to the calculated and directly measured datasets for graphical presentation of the concentration-dependent responses. These polynomial regressions were not used to calculate the transferred OD_{750} values.

S 2.4.1 Pressure Measurement as Indicator of Metabolic Activity

Gas formation during cultivation leads to an increase in internal pressure within the sealed serum bottles. Headspace pressure was therefore used as an indicator of metabolic activity. At the end of the cultivation period, bottle pressure was measured while maintaining the bottles at cultivation temperature (39 °C) to avoid temperature-dependent pressure fluctuations. A sterile BD Microlance™ cannula was inserted through the butyl rubber stopper and connected via neoprene tubing (Watson Marlow) and a 0.22 µm sterile filter (Minisart NML, Sartorius) to either a vacuum gauge (G 1111-VAC, Greisinger) or a digital barometer (GDH 14 AN, Greisinger). Pressure values are reported as overpressure relative to ambient atmospheric pressure (Δp, bar). Measurements were performed immediately prior to gas sampling to ensure consistency between pressure and gas composition analysis.

S 2.4.2 Gas Chromatography for Gas Phase Analysis

Headspace gas composition was determined at the end of each cultivation period using a 3000 Micro GC System (INFICON Holding AG) equipped with a thermal conductivity detector (TCD). Prior to sampling, serum bottles were equilibrated at cultivation temperature (39 °C) in a temperature-controlled water bath (ONE 10, Memmert GmbH + Co. KG) to minimize temperature-dependent variability in gas solubility and pressure. A gas sample of 3–5 mL was withdrawn through the butyl rubber stopper using a sterile syringe and introduced into the µGC

system, which automatically aspirated the sample at a rate of 10 mL min^{-1} using its internal vacuum pump. Gas separation was performed using a two-channel configuration. Channel A consisted of a PLOTU pre-column coupled to an Rt-Molsieve 5Å main column (Agilent) for quantification of hydrogen (H_2), nitrogen (N_2), and oxygen (O_2), using helium as carrier and reference gas at an operating pressure of 1.72 bar. Channel B employed an Rt-Q-Bond column (Agilent) for carbon dioxide (CO_2) analysis at an operating pressure of 1.38 bar. The injection system, valves, and columns were maintained at 80 °C throughout analysis. Quantification was performed based on peak area integration. Instrument calibration was conducted using a seven-point standard gas mixture covering the expected concentration ranges of hydrogen and carbon dioxide. This analytical setup enabled reproducible and simultaneous determination of headspace hydrogen, carbon dioxide, and nitrogen concentrations under all experimental conditions.

S 2.4.3 High-Performance Liquid Chromatography for Liquid Metabolite Analysis

Liquid-phase metabolites were quantified at the end of each cultivation period using high-performance liquid chromatography (HPLC). From each serum bottle, 2 mL of culture supernatant were withdrawn through the butyl rubber stopper using a sterile BD Microlance™ cannula and transferred into 2 mL microcentrifuge tubes. Samples were centrifuged for 10 minutes at 13,000 rpm using a Heraeus™ Pico 17 centrifuge (Thermo Fisher Scientific) to remove suspended particles and residual fungal biomass. Subsequently, 1 mL of the clarified supernatant was filtered through a 0.22 µm cellulose acetate syringe filter (Restek) and transferred into chromatography vials (Brown Chromatography Supplies) sealed with Teflon-lined caps. Metabolite analysis was performed on an Agilent 1100 HPLC system equipped with a Rezex ROA Organic Acid H^+ (8%) guard column (50 mm × 7.8 mm) and a corresponding analytical column (300 mm × 7.8 mm, 8 µm particle size; Phenomenex Ltd.). The mobile phase consisted of 5 mM sulfuric acid (H_2SO_4) delivered at a flow rate of 0.5 mL min^{-1}. The total run time was 40 minutes. The column compartment and refractive index detector (RID) were maintained at 50 °C to ensure stable baseline conditions. In addition to RID detection, a variable wavelength detector (VWD) set to 220 nm was used for compound-specific absorbance monitoring. Quantification was performed using external calibration curves generated from analytical standards of acetate, ethanol, citrate, succinate, formate, lactate, malate and glucose in the concentration range of 0.1–10 g L^{-1}. Peak areas were integrated using instrument-specific software, and metabolite concentrations were calculated based on linear regression of the respective calibration curves. All calibration curves exhibited coefficients of determination (R^2) $\geq$ 0.995. This analytical procedure enabled reproducible quantification of major fermentation products and residual carbon sources across all experimental conditions.

Further information

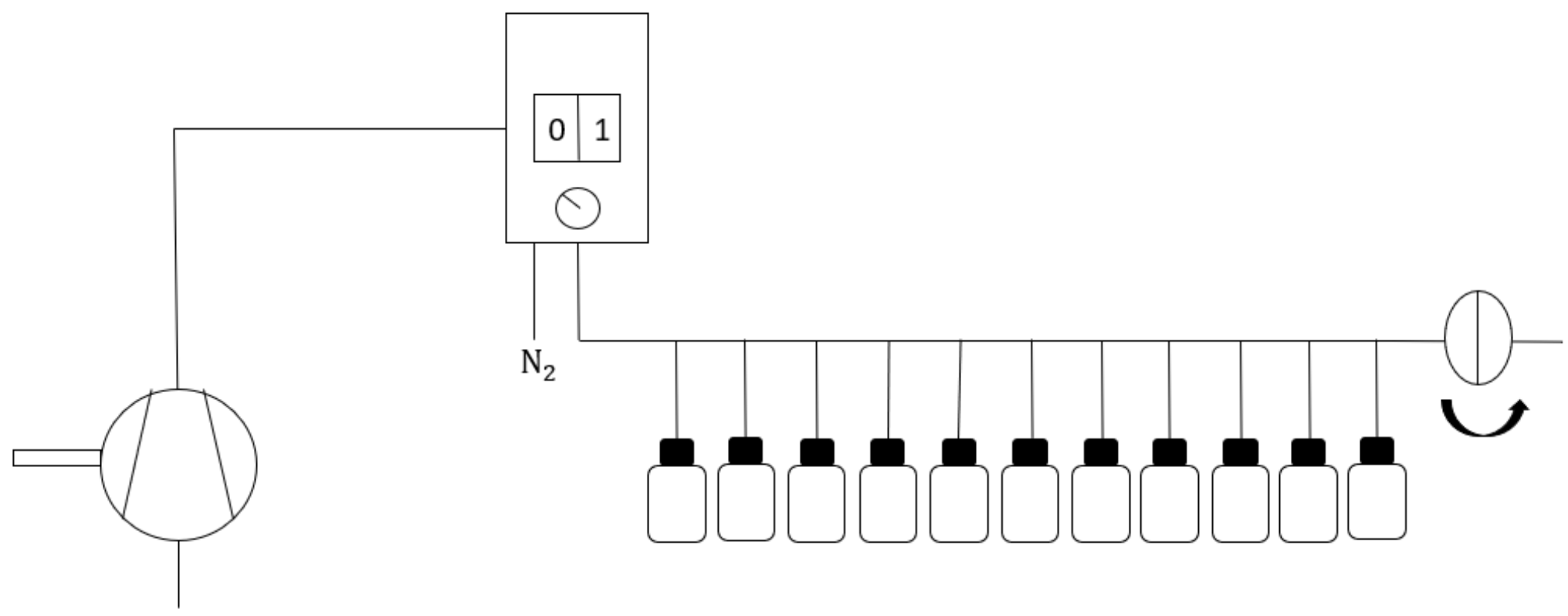

Figure S1: Schematic representation of the custom-built anaerobization station used for N_2-based cultivation. The system enables sequential evacuation and nitrogen overpressure cycles of sealed serum bottles to establish anaerobic conditions prior to incubation.

Buffer Screening

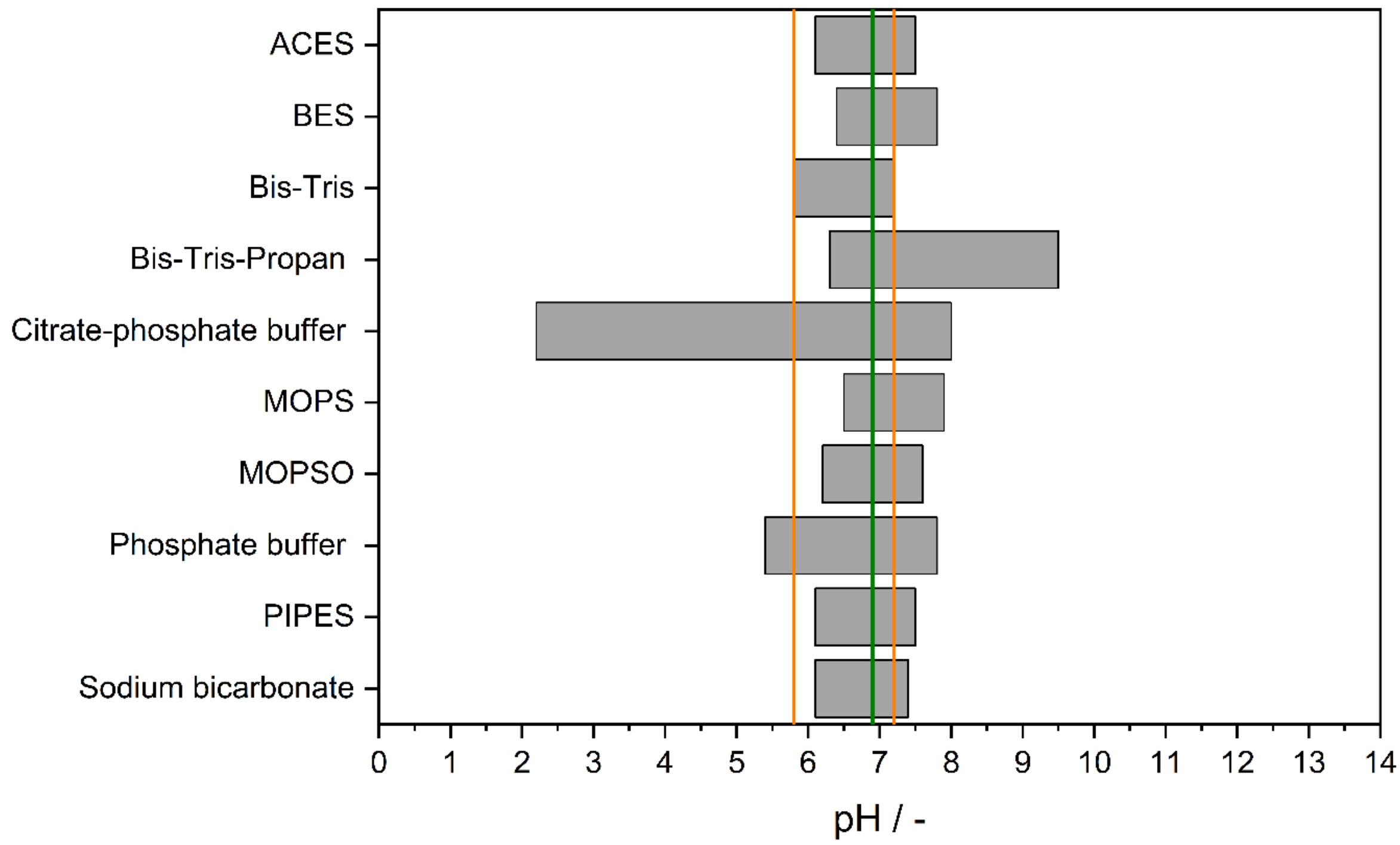

Figure S2: Buffering ranges of the selected non-carbonate buffer systems across the investigated concentration series. Orange lines indicate the physiological rumen pH range, and the green line denotes the commonly reported pH optimum for anaerobic gut fungi cultivation.

Table S1: Overview of buffer–concentration combinations evaluated in the initial buffer screening under N_2 atmosphere. Numbers indicate experimental conditions used for subsequent biological evaluation. Only buffer systems maintaining post-sterilization pH values within the defined stability range (6.9 ± 0.1) were selected for further cultivation experiments.

Concentration [g/L]	0,5	1,0	2,0	4,0	6,0	8,0	10
ACES		1	2	3	4	5	6
BES				7	8	9	10
Bis-Tris			11	12	13	14	15
Bis-Tris-Propan				16	17		
Citrate-phosphate buffer					18		
MOPS	19	20	21	22	23	24	25
MOPSO		26	27	28	29	30	31
Phosphate buffer		32	33	34	35	36	37
PIPES					38		
Sodium bicarbonate		39					

However, because autoclaving and the establishment of anaerobic conditions can induce substantial pH shifts, a preliminary stability screening was performed prior to biological evaluation. In this initial experiment, the pH of all candidate buffer systems was measured after the establishment of anaerobic conditions and autoclaving to determine whether the target pH (6.9 ± 0.1) could be maintained under cultivation conditions. The results of this physicochemical stability assessment are presented in **Fehler! Verweisquelle konnte nicht gefunden werden.**. Only buffer systems that exhibited stable post-sterilization pH values were subsequently selected for fungal cultivation experiments. **Fehler! Verweisquelle konnte nicht gefunden werden.** summarizes the post-sterilization pH measured for each buffer across the tested concentration series (0.5–10 g L^{-1}). The two red lines indicate the acceptance window used in this study (pH 6.9 ± 0.1). Across most Good's-type zwitterionic buffers (e.g., MOPS, MOPSO, BES, PIPES, ACES, Bis-Tris) and the citrate–phosphate and phosphate systems, the post-sterilization pH remained close to the target range over large parts of the concentration series, indicating that these buffers maintain target pH values under the applied anaerobic and sterilization conditions. This behavior is

consistent with the design goals of Good's buffers, which were developed to provide buffering with minimal chemical reactivity [17].

In contrast, sodium hydrogen carbonate showed a pronounced concentration-dependent alkaline shift after establishment of anaerobic conditions withN_2 /autoclaving, with pH increasing progressively at higher $NaHCO_3$ levels. This is expected for a volatile CO_2/HCO_3^- system: removal of CO_2 from the gas phase (here by N_2 exchange) perturbs the carbonate equilibrium and changes the effective buffer speciation, causing pH drift rather than stable control [35, 36]. Similar sensitivity of CO_2/HCO_3^- systems, and the risk of introducing pH artefacts when pH is not continuously regulated, has been emphasized in broader guidance on buffering regimes [37]. Notably, Bis-Tris-Propan buffer also displayed an alkaline shift at high concentrations, indicating that pH stability must be empirically validated for each buffer under the exact anaerobic/sterilization workflow rather than assumed *a priori*. For the subsequent biological evaluation of pH-stable buffer conditions, *N. cameroonii* G341 was used as model strain due to its robust growth and consistent hydrogen production in the initial atmosphere screening (**Fehler! Verweisquelle konnte nicht gefunden werden.**), enabling sensitive detection of growth loss under carbonate-free conditions.

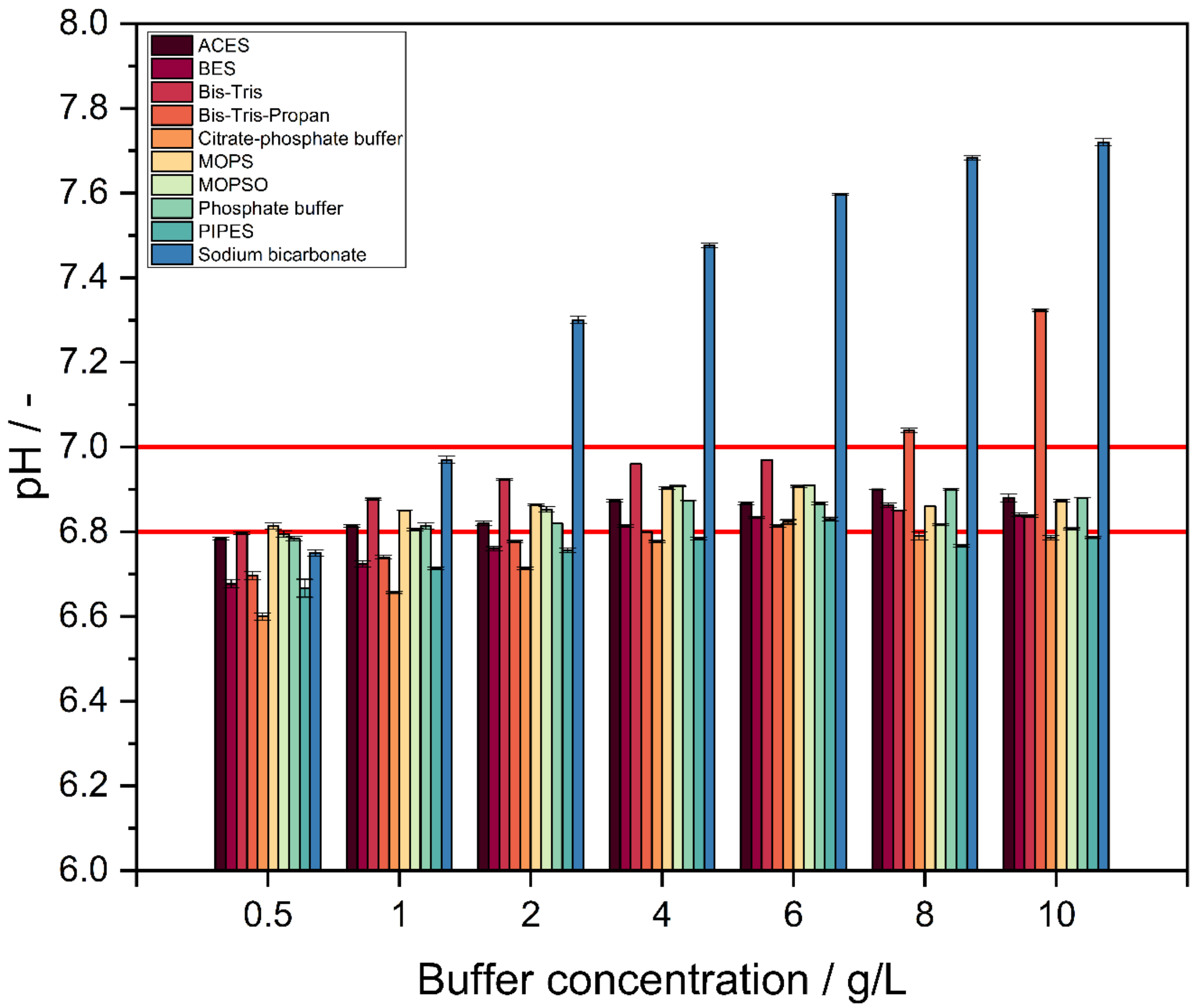

Figure S3: Post-sterilization pH values of the indicated buffer systems after the establishment of anaerobic conditions under 100% N_2 and autoclaving (121 °C, 20 min) across the tested concentration range (0.5–10 g L^{-1}; n = 3). Red lines denote the predefined acceptance window for pH stability (6.9 ± 0.1). Sodium hydrogen carbonate exhibits a concentration-dependent alkaline shift following N_2 treatment, whereas several non-carbonate buffer systems maintain pH values within the target range.

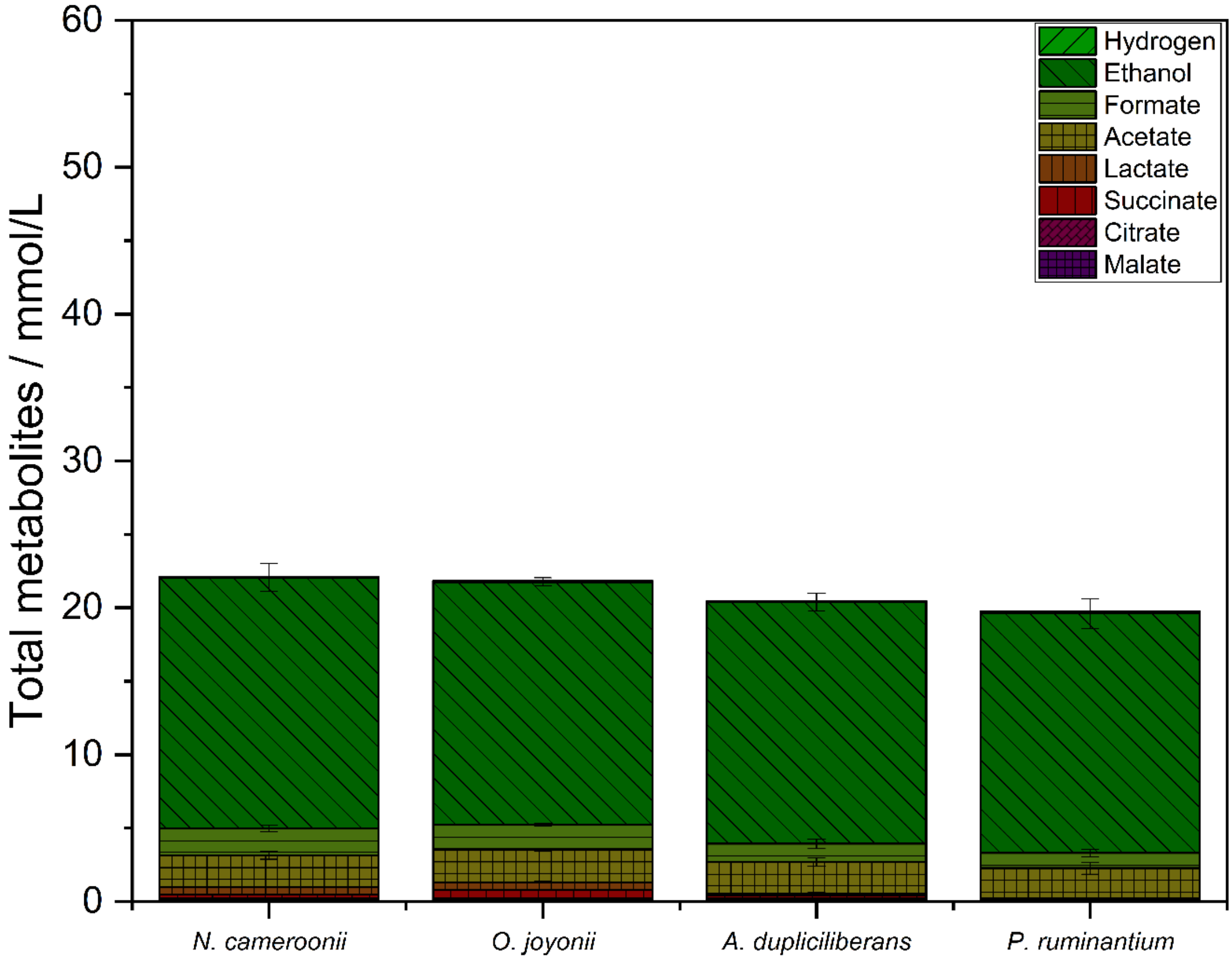

Figure S4: Effect of sodium chloride supplementation on the metabolic activity of anaerobic gut fungal strains under a 100% N_2 atmosphere. NaCl was added with the same molar Na^+ concentration as 6 g L^{-1} $NaHCO_3$ used in the standard cultivation medium. NaCl supplementation did not restore hydrogen production or sustained metabolite formation. Data represent biological triplicates; error bars indicate standard deviation.

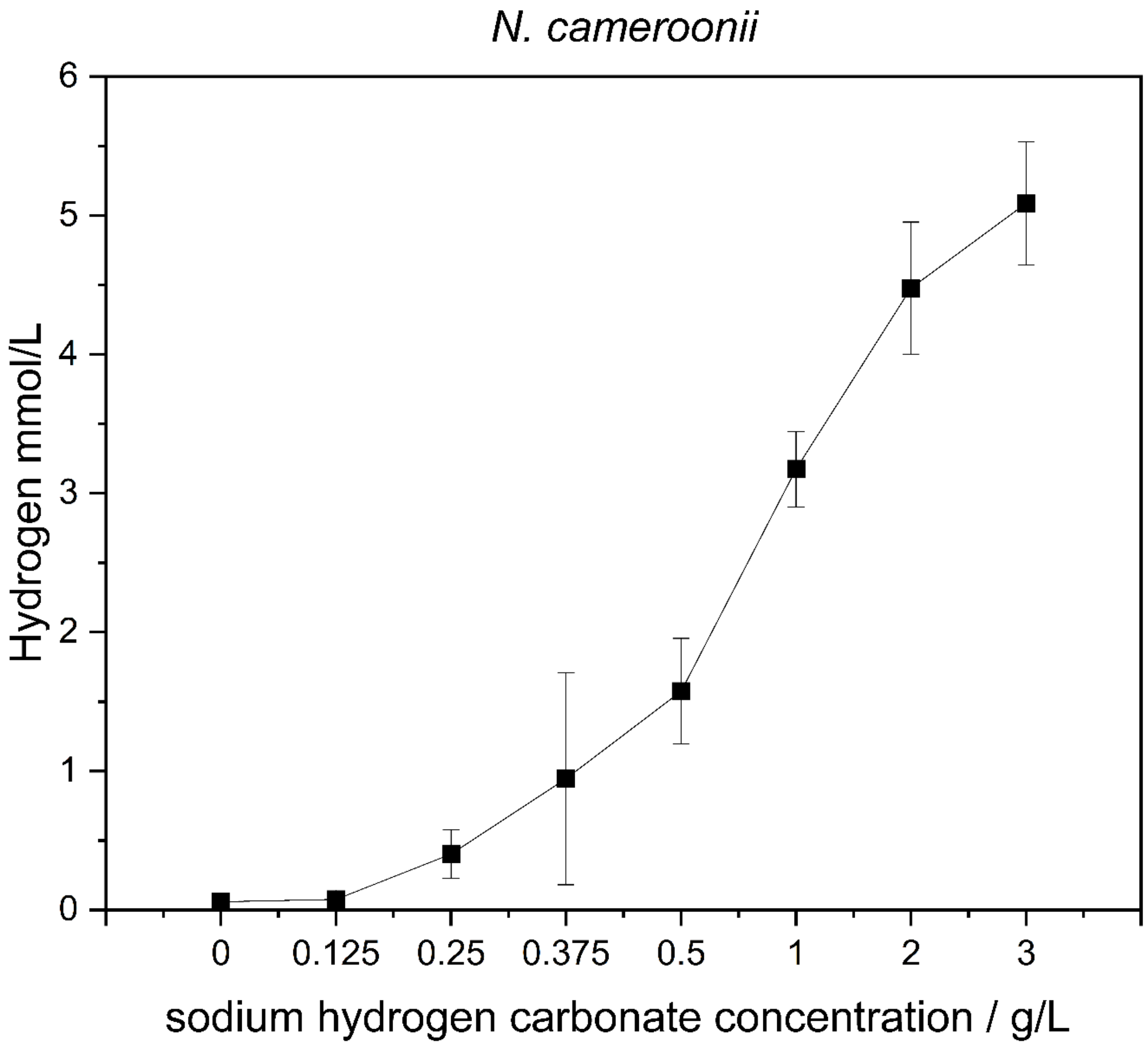

Figure S5: Hydrogen production of N. cameroonii during the minimal sodium carbonate experiment under 100% N_2 atmosphere with increasing sodium hydrogen carbonate concentrations (0–3 g L^{-1}). Hydrogen formation is shown separately as exemplary to highlight plateau like behavior. H_2 production increased strongly between 0.25 and 2 g L^{-1} $NaHCO_3$ and showed smaller incremental gains between 2 and 3 g L^{-1}, indicating an approach toward saturation rather than a fully resolved plateau within the tested concentration range. Data represent biological triplicates (n = 3); error bars indicate standard deviation.

Screening of carboxylic acids

Table 2: Candidate compounds added in the carboxylic screening.

Tested compound	Chemical form used	Compound class	Supplier
Abietic acid	Free acid	Resin acid / diterpenoid carboxylic acid	Carl Roth GmbH + Co. KG
Acetoacetic acid benzyl ester	Ester	β-keto ester	Sigma-Aldrich Chemie GmbH
Acrylic acid	Free acid	Unsaturated monocarboxylic acid	Sigma-Aldrich Chemie GmbH
Adipic acid	Free acid	Saturated dicarboxylic acid	Sigma-Aldrich Chemie GmbH
Succinic acid	Free acid	Saturated dicarboxylic acid	Carl Roth GmbH + Co. KG
Benzoic acid	Free acid	Aromatic monocarboxylic acid	Carl Roth GmbH + Co. KG
Butyric acid	Free acid	Short-chain monocarboxylic acid	Carl Roth GmbH + Co. KG
Pyruvic acid	Free acid	α-keto acid	Carl Roth GmbH + Co. KG
Citric acid	Free acid	Tricarboxylic acid	Carl Roth GmbH + Co. KG
Fumaric acid	Free acid	Unsaturated dicarboxylic acid	Carl Roth GmbH + Co. KG
Gibberellic acid	Free acid	Diterpenoid carboxylic acid	Carl Roth GmbH + Co. KG
Glutaric acid	Free acid	Saturated dicarboxylic acid	Sigma-Aldrich Chemie GmbH
Levulinic acid	Free acid	γ-keto acid	Carl Roth GmbH + Co. KG
Maleic acid	Free acid	Unsaturated dicarboxylic acid	Sigma-Aldrich Chemie GmbH
Malonic acid	Free acid	Saturated dicarboxylic acid	Sigma-Aldrich Chemie GmbH
Oleic acid	Free acid	Long-chain unsaturated fatty acid	Sigma-Aldrich Chemie GmbH
Oxalic acid	Free acid	Dicarboxylic acid	Carl Roth GmbH + Co. KG
Salicylic acid	Free acid	Aromatic hydroxycarboxylic acid	Carl Roth GmbH + Co. KG
L-Proline	Amino acid	Amino acid / carboxyl-containing compound	Carl Roth GmbH + Co. KG
Sodium hydrogen carbonate	Sodium salt	Inorganic carbon positive control	Carl Roth GmbH + Co. KG

Validation of the OD_{750} biomass calibration

While the preceding experiments used metabolic output as a proxy for growth, interpretation of fermentation patterns alone does not allow discrimination between altered metabolic flux distribution and genuine differences in biomass formation. Because anaerobic gut fungi exhibit complex morphologies and substrate-associated growth, reliable biomass quantification represents a methodological challenge. To address this limitation, an OD_{750}-based biomass detection approach calibrated against defined dry weight standards and an inert activated carbon reference was established and validated. In contrast to previous mechanistic assays focusing primarily on filamentous model strains, the calibration procedure included all six fungal strains to assess inter-strain variability in light scattering behavior and to evaluate transferability across morphotypes. The comparison between activated-carbon-transferred and directly measured OD_{750} values is shown in Figure 8. Activated carbon produced concentration-dependent optical responses on both photometric systems, but the magnitude of the response differed between instruments. Therefore, fungal OD_{750} values measured on Photometer 1 were transferred separately at each concentration by multiplying them by the corresponding ratio between the activated carbon responses of Photometer 2 and Photometer 1. The resulting calculated values were then compared with fungal biomass standards measured directly on Photometer 2. Agreement between the calculated and directly measured values was concentration- and strain-dependent. Larger relative deviations were observed particularly at the lowest biomass concentrations, where the OD_{750} values were close to the instrumental baseline and small absolute differences resulted in comparatively large percentage deviations. Differences among strains additionally reflect their distinct aggregation, branching, and resuspension behavior. These results show that activated carbon can be used as an inert optical reference for approximate transfer of OD_{750} values between photometric systems within the experimentally tested concentration range. However, the method should be regarded as a comparative biomass proxy rather than a substitute for direct, strain- and instrument-specific calibration, particularly at very low biomass concentrations. This likely explains the larger deviations observed at the extremes of the calibration range and

underscores that strain-specific optical behavior remains relevant in morphologically heterogeneous AGF cultures [11].

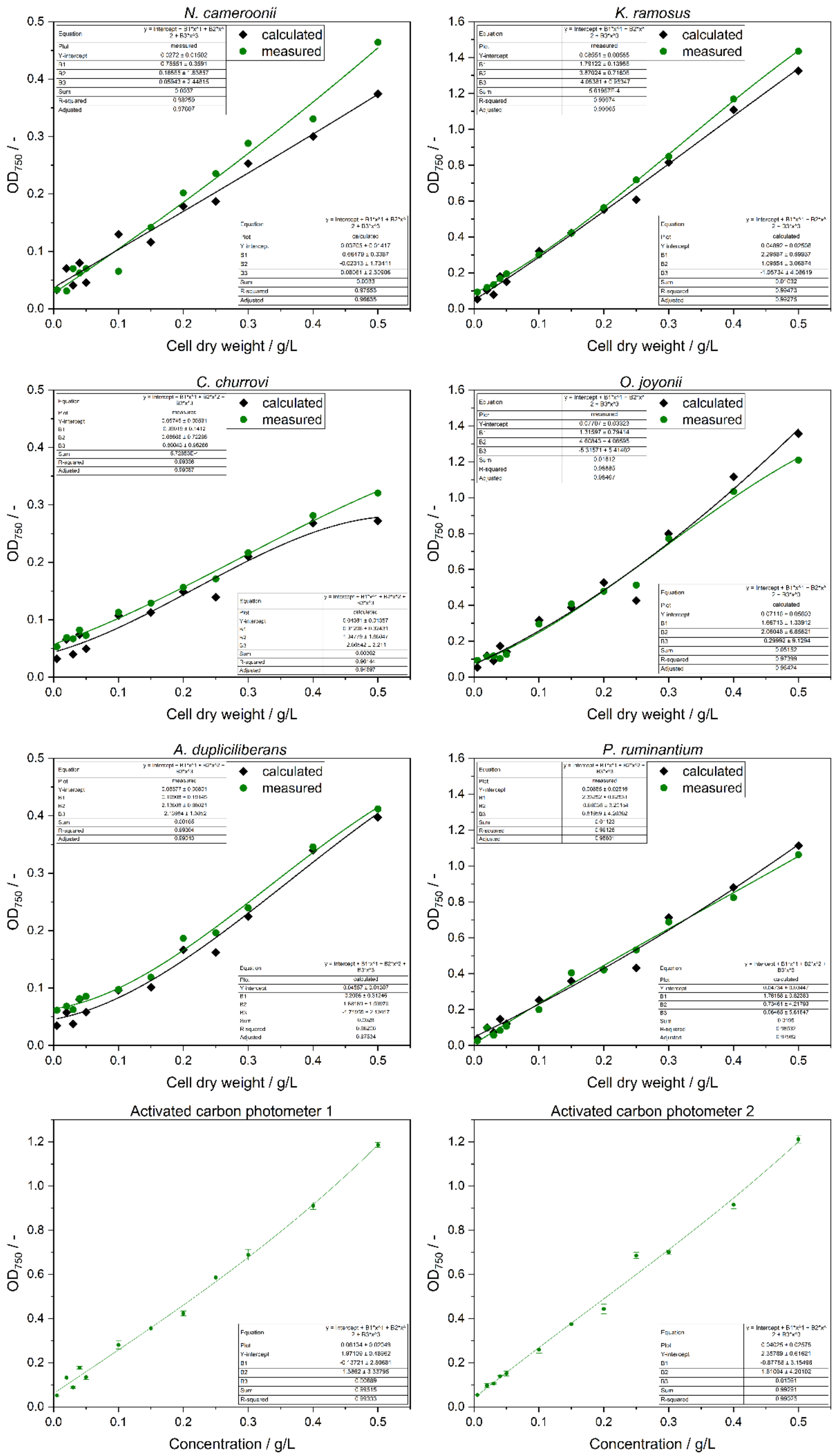

N. cameroonii
K. ramosus
C. churrovi
O. joyonii
A. dupliciliberans
P. ruminantium
Activated carbon photometer 1
Activated carbon photometer 2
calculated
measured
OD_{750} / -
Cell dry weight / g/L
Concentration / g/L

Figure S6: Comparison of directly measured and activated-carbon-transferred OD_{750} values for fungal biomass on Photometer 2. In the six upper panels, green circles represent OD_{750} values measured directly for fungal biomass suspensions of known dry-mass concentration (0.005–0.5 g L^{-1}) on Photometer 2, whereas black diamonds represent values transferred from Photometer 1 at each matched concentration according to OD(s,P2,c) = OD(s,P1,c) × [OD(AC,P2,c) / OD(AC,P1,c)]. Here, s denotes the fungal strain, c the dry-mass concentration, AC activated carbon, and P1 and P2 Photometers 1 and 2, respectively. The two lower panels show the activated carbon OD_{750} responses measured on Photometers 1 and 2 at the same concentrations. Third-order polynomial lines are included only to visualize the concentration-dependent datasets and were not used to calculate the transferred values. Direct fungal and activated carbon measurements represent means of ten technical replicates; error bars indicate standard deviation.
